# Efficient and rapid cell surface functionalization: a sub-minute selenol-yne click reaction for bioconjugation

**DOI:** 10.1039/d5sc05541e

**Published:** 2025-10-29

**Authors:** Fangjian Shan, Xingyu Heng, Lihua Yao, Guichuan Xu, Jun Hu, Xiangqiang Pan, Gaojian Chen

**Affiliations:** a State and Local Joint Engineering Laboratory for Novel Functional Polymeric Materials, Jiangsu Key Laboratory of Advanced Functional Polymer Materials, College of Chemistry, Chemical Engineering and Materials Science, Soochow University Suzhou 215123 P. R. China panxq@suda.edu.cn gchen@suda.edu.cn; b School of Chemistry and Chemical Engineering, Southeast University Nanjing 211189 P. R. China

## Abstract

Cell surface engineering plays a crucial role in enhancing the functionality and therapeutic potential of living cells through precise and controllable surface modifications. However, traditional conjugation strategies often suffer from limited efficiency or inherent complexity. In this work, we developed a selenol-yne click (SYC) reaction for efficient and rapid cell surface functionalization, enabling covalent attachment of diverse payloads to a variety of cell types. The SYC reaction enables highly efficient conjugation of fluorescent molecules, micron-scale “backpack” particles, and functional polymers onto cell surfaces, including HeLa, B16-OVA, and Jurkat T cells. Drug-loaded backpacks carried by the cells demonstrated effective drug delivery to target cells. Moreover, cell surface glycosylation *via* this method enables efficient modulation of cell–cell interactions and immune responses. Importantly, glycosylation was also successfully achieved in living zebrafish through the SYC reaction, demonstrating its potential for *in vivo* applications. This SYC-based strategy offers a fast and versatile platform for advancing cell surface engineering.

## Abstract 2

A rapid selenol-yne click (SYC) reaction enables efficient and versatile cell surface engineering, allowing covalent attachment of diverse molecular and material payloads to live cells through fast SeH–alkyne coupling on a short timescale.

## Graphical abstract

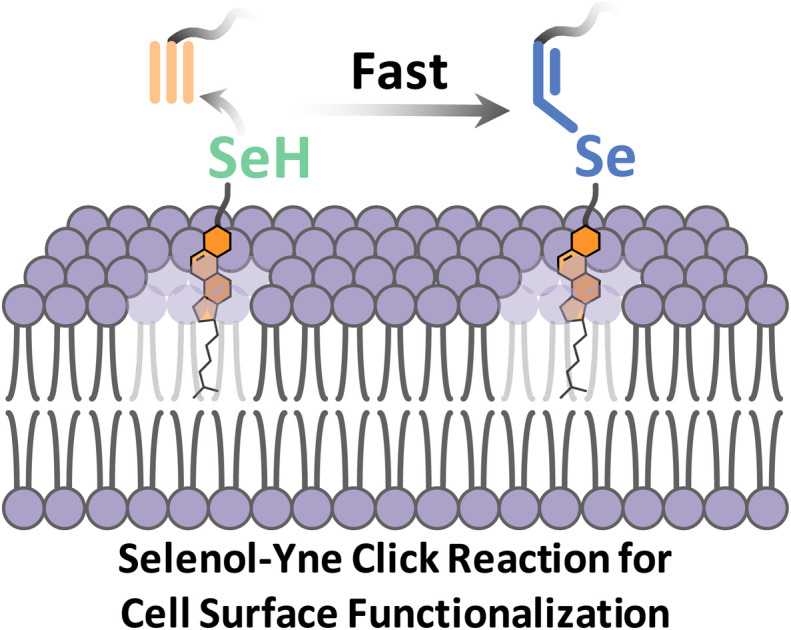

## Introduction

Cell surface engineering has emerged as a powerful tool in various fields, including diagnostics, therapeutics, and fundamental cell biology research.^1–8^ The capability to selectively modify and functionalize the exterior of living cells with natural or synthetic materials, such as nanoparticles, enables targeted drug delivery, improved cell imaging, and the modulation of intercellular interactions to boost immune responses.^9–13^ The precise control offered by cell surface functionalization enables the meticulous manipulation of cellular behavior and the development of advanced biohybrid systems.

Significant progress has been made in bioconjugation chemistry.^11,14–17^ However, a major challenge persists in achieving rapid and efficient modification of cell surfaces without compromising cellular viability and functionality. Bioorthogonal methods facilitate the introduction of non-native functional groups, such as azides, onto mammalian cell surfaces through metabolic engineering, thereby enabling surface modification *via* azide–alkyne reactions.^18,19^ Alternatively, disulfide bonds can be cleaved using TCEP, and the thiol–maleimide reaction provides an efficient surface engineering strategy.^20^ However, the disruption of disulfide bonds may adversely affect the functionality of membrane proteins. Many established bioconjugation strategies, including those utilizing amine-reactive NHS esters,^21^ amino-yne^22^ or azide–alkyne coupling,^23,24^ often require reaction times on the order of hours for cell surface modification. Such prolonged exposure to potentially reactive reagents can result in off-target modifications, induce cellular stress, and compromise cell integrity, particularly in delicate or primary cell types. Furthermore, *in vivo* applications, rapid conjugation can enhance targeting specificity and reduce the circulation time of unbound reagents, thereby improving therapeutic efficacy and minimizing adverse effects.

**Scheme 1:**
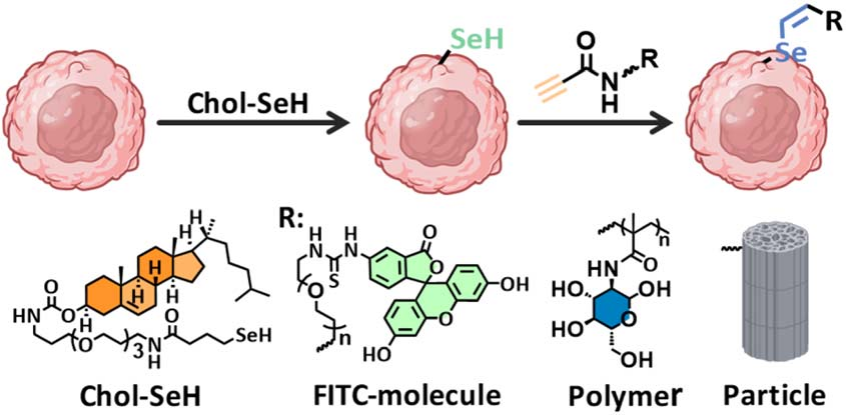
SYC for efficient and rapid bioconjugation on cell surface.

To address these limitations, the development of bioorthogonal reactions that proceed with exceptional speed and selectivity under physiological conditions is highly desirable. Click chemistry, characterized by its high yield, mild reaction conditions, and tolerance to biological environments, has revolutionized bioconjugation.^25–28^ However, even within the realm of click reactions, achieving sub-minute reaction kinetics on complex cell surfaces in a facile manner remains a formidable challenge. The selenol-yne click (SYC) reaction has emerged as an exceptionally fast conjugation strategy, enabling the rapid formation of responsive C–Se bonds within minutes.^29–31^ Although its potential for ultrafast coupling is recognized, its application in the complex and dynamic environment of living cell surfaces remains largely unexplored. Herein, we present the first demonstration of a highly efficient SYC for sub-minute bioconjugation on live cells (Scheme 1). To avoid the impact of covalent modifications on the native functions of cell surface molecules, we introduce the selenol moiety onto the cell membrane *via* cholesteryl selenol (Chol–SeH), a lipophilic anchor that facilitates membrane integration. Simultaneously, we prepare a diverse array of alkynyl functional entities ready for conjugation, including fluorescent molecules, sugar-based polymers, and nanoparticles. By leveraging the remarkable reactivity of the SYC, we achieve rapid and robust conjugation of these functional entities to various cell types within minutes, significantly surpassing the reaction rates of existing bioconjugation methods. Furthermore, the potential of the SYC coupling method for *in vivo* applications has been demonstrated. This unprecedented speed, coupled with the versatility demonstrated through the conjugation of diverse payloads, underscores the potential of this selenol-yne click strategy to revolutionize cell surface engineering, enabling more precise, efficient, and minimally invasive manipulation of living cells for a wide range of biological applications.

## Results and discussion

**Fig. 1:**
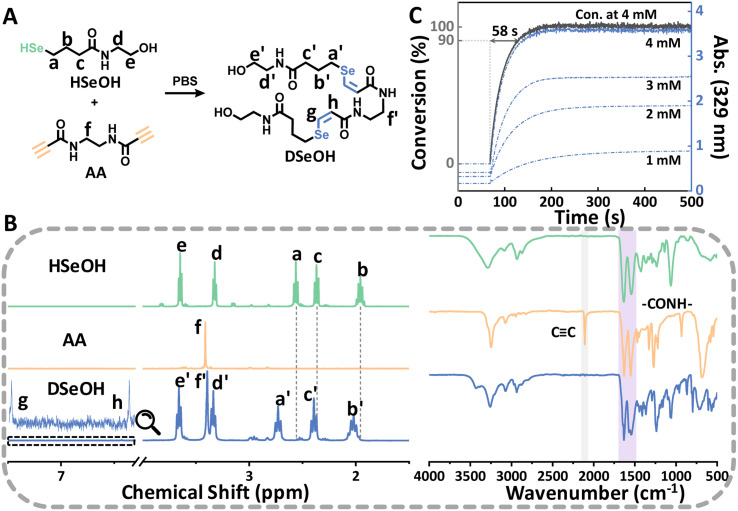
(A) Model SYC in PBS and (B) ^1^H NMR/IR spectra of reactant and product obtained in deuterated phosphate buffer; (C) absorbance-time curves (blue lines) of the reaction system at different initial concentrations of AA, and the conversion curve (grey line) at an AA initial concentration of 4 mM. HSeOH (2 equiv.) was reacted with AA at room temperature.

To illustrate the feasibility of the reaction under physiological conditions, we constructed a reaction model of HSeOH and AA in deuterated phosphate buffer (PBS/D_2_O) (Fig. 1A). After mixing HSeOH and AA, the ^1^H NMR spectrum was recorded immediately (Fig. 1B, left). The spectrum shows that the characteristic peaks of HSeOH and AA are barely detectable, whereas the peaks corresponding to the double bonds (g and h) in DSeOH—the addition product formed between selenol and alkyne—are clearly observed. Deuteration of the double-bond hydrogens, caused by the deuteration of selenol and alkyne hydrogens, results in weak signals. To observe a clear double bond peak, see the NMR spectrum of DSeOH obtained in non-deuterated buffer in the SI. Notably, the addition product exhibits a high degree of *Z*/*E* selectivity, with a *Z*/*E* ratio of 65 : 1. Under base-free conditions, we propose that this addition reaction proceeds similarly to the amino-yne addition, involving an intramolecular proton transfer process (Fig. S1).^32^ DFT calculations were conducted to evaluate the transition state free energies for both the *E* and *Z* pathways. The results indicate that the pathway leading to the *Z*-isomer has a lower activation barrier, making the reaction more favourable *via* this route. The disappearance of the triple bond signal (2109 cm^−1^) in the infrared spectrum (Fig. 1B, right), along with the four peaks (1646, 1632, 1570, 1543 cm^−1^) in the amide region and the mass spectrometry results (see SI), further confirm the successful progress of the reaction. In addition, even with an excess of selenol, the reaction with the alkyne rarely proceeded to the double addition within the investigated time frame (Fig. S2). AA and DSeOH exhibit UV-Vis absorption at 275–450 nm (Fig. S3A), allowing the concentrations of reactants and products in the reaction system to be monitored in real time using a UV-Vis spectrophotometer, thereby enabling the construction of kinetic curves (Fig. 1C). In PBS, the SYC proceeds at an extremely fast rate, reaching 90% conversion in just 58 seconds at an initial AA concentration of 4 mM, which is faster than most previously reported conjugation reactions.^33–36^ For example, the classic cycloalkyne–azide reaction typically requires approximately 30 minutes. Potential side reactions arising from nonspecific interactions between alkynes and native amino or thiol groups on the cell surface may affect the efficiency and selectivity of the SYC reaction.^37^ To investigate this, we evaluated the reaction rates of alkynes with different nucleophilic groups and compared their selectivity in buffered solutions containing selenol, amino, and thiol groups (Fig. S4 and S5). The results demonstrate that the ultrafast kinetics of the SYC reaction account for its high efficiency and selectivity.

**Fig. 2:**
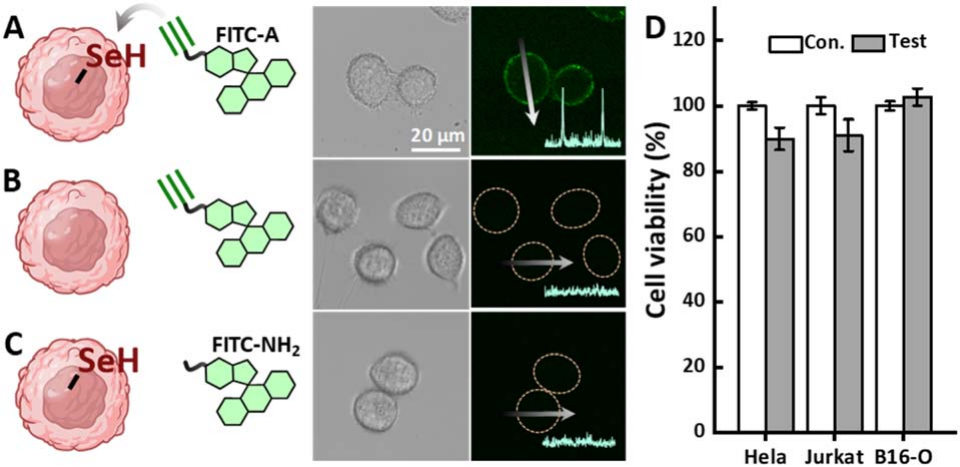
SYC for HeLa cells surface modification with FTIC. Confocal images and fluorescence line scan analysis of HeLa cells after 10 minutes of incubation with Chol–SeH (A, C) or cholesterol (B) and 15 minutes staining with FITC-A (A, B) or FITC-NH_2_ (C) and (D) cell viability assay of the SYC reaction-based cell surface engineering method on HeLa, B16-OVA, and Jurkat cells. The error bars represent mean ± SD (*n* = 6).

A fluorescent dye with an alkyne functional group (FITC-A) was used to assess the modification efficiency on the cell surface. The fluorescence signal was uniformly distributed on the cell surface after 15 minutes staining with FITC-A, with no signs of internalization (Fig. 2A). Only when the cell surface and the fluorescent molecule are properly selenylated and alkyned, respectively, will the cell surface be properly modified and show fluorescent signals (Fig. 2B and C). The SYC-based cell surface functionalization method exhibits the selectivity of bioorthogonal reactions. At the same time, Cell Counting Kit-8 (CCK-8) assays showed that the method exhibited no significant cytotoxicity (Fig. 2D).

**Fig. 3:**
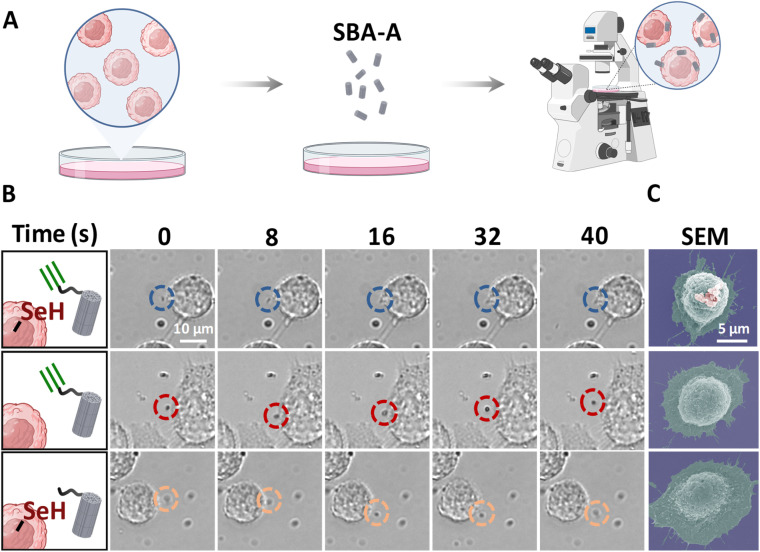
SYC for loading cellular backpacks onto HeLa cells. (A) Cell image tracking to visualize the interaction between SBA material and HeLa cells; (B) SBA-A was conjugated onto the surface of HeLa cells within one minute *via* SYC, compared with controls lacking selenol or alkyne groups; (C) SEM images of HeLa cells loaded with cellular backpacks *via* SYC and the failed loading due to the absence of selenol or alkyne groups.

Drug-loaded engineered microparticles attach to the cell surface like backpacks, effectively regulating cell behavior.^38–40^ The stability of the connection between the particles and cells is crucial to the effectiveness of these “cellular backpacks”.^38^ We explored the use of SYC as a connection method between cells and particles to support cellar backpack attachment. The interaction between particles and cells was continuously tracked using microscopic imaging (Fig. 3A). The SYC efficiently anchors alkynylated mesoporous silica (SBA-A) to the cell surface (Fig. 3B). Notably, this process is completed within seconds, primarily determined by the diffusion rate of the particles toward the cell membrane. In contrast, for the control group lacking alkynyl or selenol functional groups, the particles, driven by Brownian motion, fail to remain on the cell surface for an extended period, ultimately leading to differences in particle attachment observed in SEM images (Fig. 3C and S6). Through the tight junctions formed by the SYC, SBA-A loaded with Nile Red (a model drug) achieves more efficient dye delivery to cells (Fig. S7). These results demonstrate that SYC enables the rapid attachment of cellular backpacks to the cell surface and promotes the effective transfer of their cargo into the cells.

**Fig. 4:**
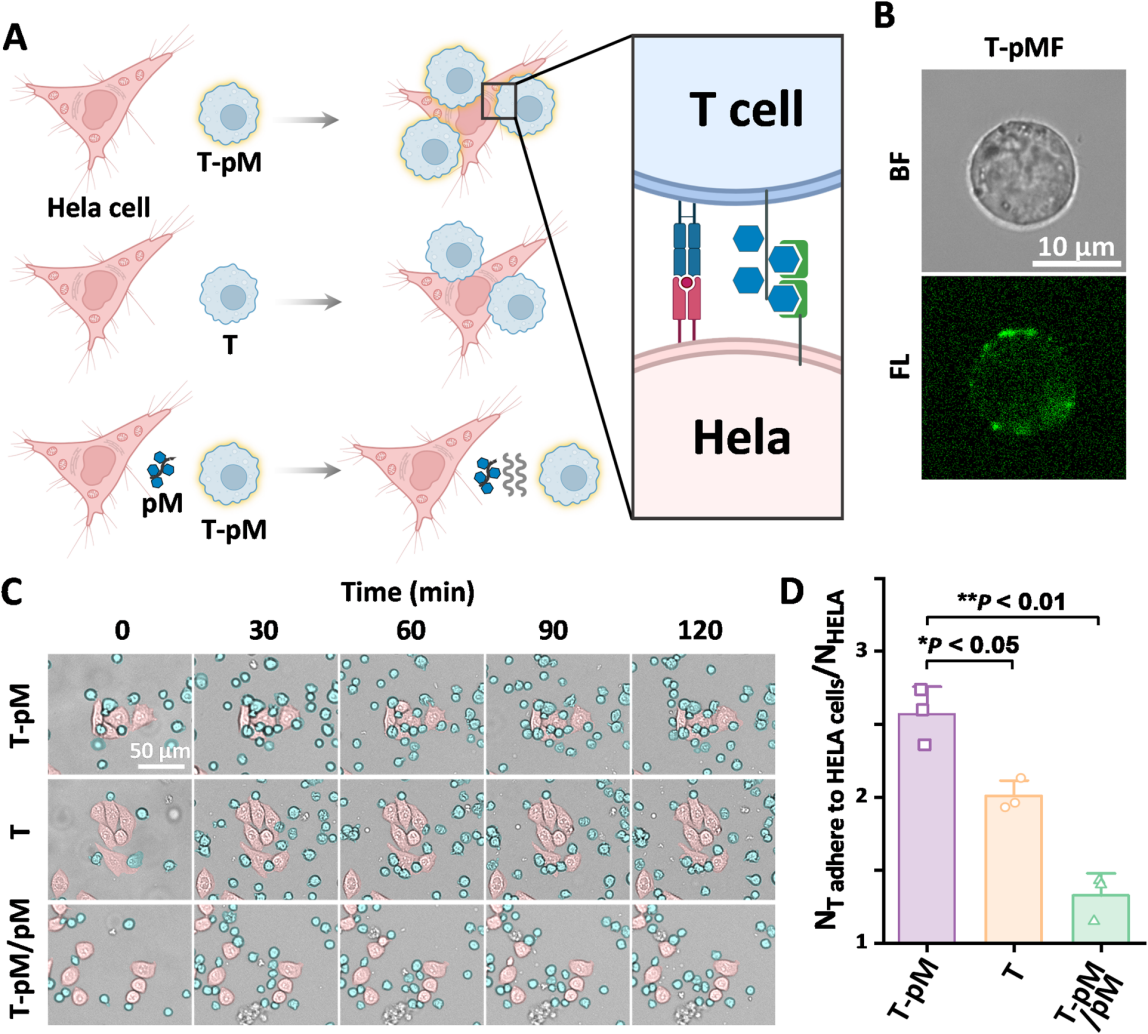
SYC for Jurkat T cells surface modification with glycopolymer. (A) Different strengths of interaction between T-pM, T, T-pM/pM and HeLa cells; (B) confocal images of T-pMF; (C) interaction relationship between T-pM, T, T-pM/pM and HeLa cells in different time periods; (D) a statistical chart of the average number of T cells adhered to HeLa cells at the 120-minutes. The error bars represent mean ± SD (*n* = 300). Statistical analysis was performed using Tukey's multiple comparison test.

Subsequently, the applicability of the method was evaluated in different cell types and application scenarios. Synthetic glycopolymers, by mimicking the multivalent effects of natural polysaccharides, interact well with glycan receptors, such as C-type lectins, *E*-selectin, and glucose transporter Glut1.^41–43^ Constructing glycopolymer layers on cell surfaces using cell surface engineering can effectively regulate glycan-mediated cell–cell interactions, thereby enhancing various immune responses, such as tumor killing and T-cell activation.^44,45^ Using the rapid SYC, we first explored its application in glycopolymer engineering of T cell surfaces to enhance their interaction with cancer cells. As show in Fig. 4A, we synthesized a glucose-based polymer with alkyne terminal groups (polyMAG-A, pM-A), which was then attached to selenol-modified T cells *via* the SYC reaction, resulting in T-pM cells. T-pM cells demonstrated significantly stronger interactions with Glut1-overexpressing HeLa cancer cells compared to unmodified T cells. This glycoengineering of T cells was verified using a fluorescently labeled analog, poly(MAG-*co*-FITC)-A (pMF-A) (Fig. 4B). To verify that the enhanced binding was due to the surface-conjugated glycopolymer, we conducted a competition assay with free pM. The free pM notably decreased the interaction between T-pM cells and HeLa cells, even below that of unmodified T cells, confirming the specific role of surface-bound pM in cancer cell recognition (Fig. 4C and D). These findings show that the glycopolymer modification of T cells *via* the SYC reaction effectively and specifically increases their binding affinity to cancer cells.

**Fig. 5:**
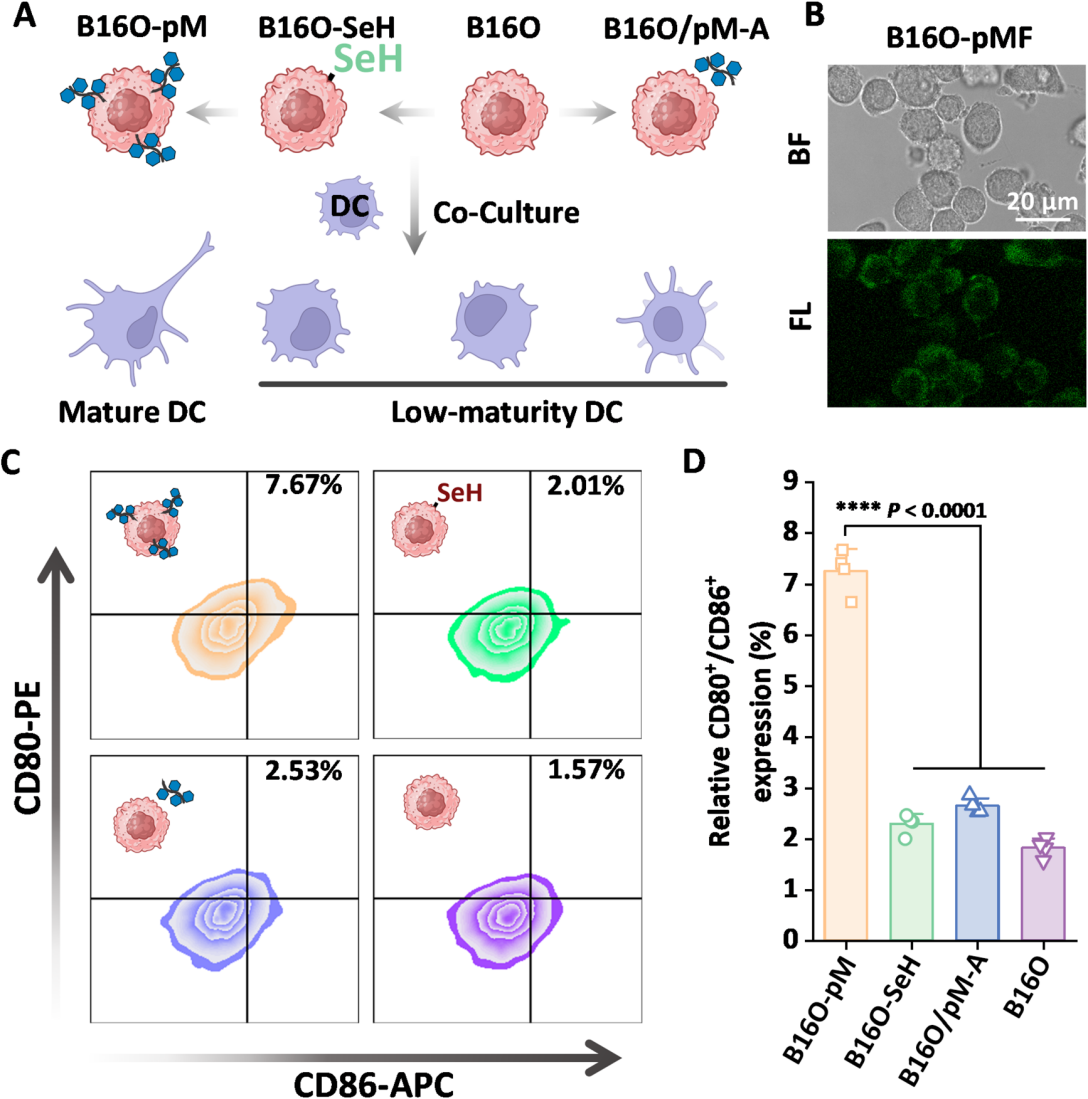
SYC for whole tumor vaccine modification. (A) Preparation of WTCVs and its immune activation on DCs; (B) confocal images of B16O-pMF; (C) flow cytograms of B16O-pM, B16O-SeH, B16O/pM-A and B16O alone on DC2.4 maturation; (D) statistical analysis of relative CD80/CD86 expression on DC2.4 incubated with different WTCVs. The error bars represent mean ± SD (*n* = 4). Statistical analysis was performed using Tukey's multiple comparison test.

We further explored the utility of the SYC method for the preparation of enhanced whole tumor cell vaccines (WTCVs) through controlled surface glycosylation. WTCVs are promising in cancer immunotherapy as they use inactivated tumor cells to present tumor antigens and damage-associated molecular patterns (DAMPs), triggering an anti-tumor immune response.^46^ Dendritic cell (DC) maturation is crucial for activating cytotoxic T cells to target cancer cells. Due to the high expression of glucose receptors like DC-SIGN on DC,^47–49^ which aid in glucose-related maturation, we proposed that glucose-based glycopolymer modification of WTCVs could enhance DC maturation. We tested this using UV-inactivated murine melanoma cells (B16-OVA, B16O) modified with the glucose-based glycopolymer (pM) *via* the SYC reaction, resulting in B16O-pM (Fig. 5A). This glycoengineering also was verified using a fluorescently labeled analog, pMF-A (Fig. 5B). We assessed the ability of glycopolymer-modified tumor cells to enhance DC maturation. As show in Fig. 5C and D, B16O-pM treatment significantly increased DC maturation compared to control groups (Chol–SeH alone, pM-A alone, or unmodified B16O), with a 3.8-fold rise over unmodified WTCVs. The pM-A treated group exhibited weak DC-stimulating activity, which can be attributed to the absence of selenol groups on the cell surface, as described in Fig. 1, preventing effective conjugation of the polymer with the cells to form B16O-pM. This demonstrates the effectiveness of the SYC reaction in functionalizing tumor cells, indicating its potential for creating advanced cancer vaccines with improved immunostimulatory capabilities.

**Fig. 6:**
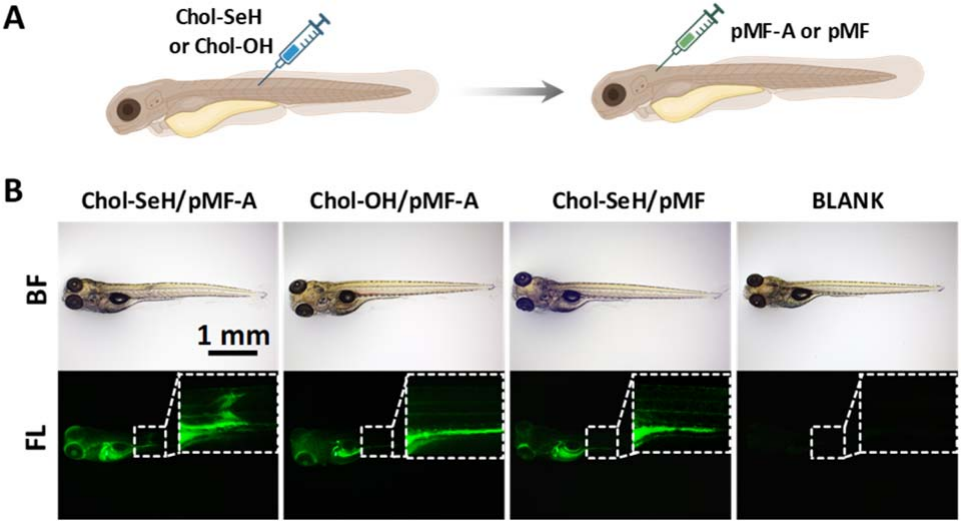
Glycosylation of zebrafish V-shaped myotomes (VSM). (A) Schematic diagram of glycan modification on VSM; (B) pMF was conjugated onto the VSM of zebrafish *via* SYC, compared with controls lacking selenol or alkyne groups.

The glycan composition of zebrafish cell membranes plays a crucial role in tissue development and regeneration.^50,51^ After establishing the practicality of the SYC conjugation reaction at the cellular level, we further applied SYC in zebrafish to assess its *in vivo* reactivity and to provide a potential tool for studying tissue glycosylation. We selected the V-shaped myotomes (VSM) as a representative tissue site because of their structural regularity and accessibility for fluorescence imaging. As shown in Fig. 6A, Chol–SeH was first microinjected into the VSM region to achieve localized selenolation, followed by injection of fluorescently labeled and alkyne-functionalized glycopolymer (pMF-A) into the common cardinal vein. One hour after injection, the glycopolymer is observed to accumulate in several regions (Fig. 6B), such as the post-ocular interstitial space, liver, and yolk sac. Meanwhile, distinct fluorescence is detected in the VSM region subjected to selenol modification, whereas no fluorescence of the glycopolymer is observed in the VSM of the control groups injected with cholesterol or alkyne-deficient glycopolymer. These results indicate that SYC can achieve glycosylation of the target tissue at the *in vivo* level.

## Conclusions

In summary, the selenol-yne click (SYC) reaction demonstrates fundamental characteristics of bioorthogonal reactions through model reactions conducted in PBS with rapid kinetics achieving completion within minutes. The successful conjugation of fluorescent molecules onto HeLa cell surfaces further validates SYC's exceptional efficiency and selectivity in biomaterial-cell surface coupling. To illustrate the broad applicability of this rapid conjugation strategy, we implemented SYC for efficient coupling of micron-scale “backpack” particles and functional polymers to diverse cell types, including adherent cells (HeLa and B16-OVA) and suspension cells (Jurkat T). The drug-loaded backpacks carried by the cells effectively delivers drug to cells. Additionally, cell surface glycosylation enabling efficient modulation of cell–cell interactions and immune functions. Importantly, SYC was successfully applied in living zebrafish, demonstrating its potential for *in vivo* applications. It should be noted that the alkynyl–carbonyl structure utilized herein exhibits excessive reactivity. While it shows good selectivity under short incubation conditions in this work, prolonged cell incubation induces unavoidable thiol-yne addition reactions, thereby compromising selectivity. Consequently, strategic modification of substituents adjacent to the alkynyl group is needed to optimize the trade-off between reaction kinetics and selectivity. In addition, appropriate alkyne design can promote a double-addition reaction in SYC, leading to the formation of C–Se bonds capable of undergoing selenoacetal exchange.^29^ The combination of rapid reactivity and dynamic exchange properties endowed by SYC holds significant potential for cell coupling and interaction regulation.^52–55^ Overall, this SYC-based surface engineering platform establishes a robust methodology for advancing cell surface modification technologies.

## Author contributions

Fangjian Shan: synthesis, characterization, data analysis, and article writing. Xingyu Heng and Lihua Yao: cell culture. Guichuan Xu and Jun Hu: theoretical guidance. Xiangqiang Pan and Gaojian Chen: conceived the core idea, supervised the whole project, and edited the manuscript.

## Conflicts of interest

There are no conflicts to declare.

## Supplementary Material

SC-016-D5SC05541E-s001

## Data Availability

The data supporting this article have been included as part of the supplementary information (SI). Supplementary information: materials, cell lines, and experimental animals; instrumentation details; experimental procedures; synthetic routes; computational details and optimized Cartesian coordinates of the molecule; IR NMR and mass spectra. See DOI: https://doi.org/10.1039/d5sc05541e.
