# Supplementary material for: Efficient and rapid cell surface functionalization: a sub-minute selenol-yne click reaction for bioconjugation
Source: Chem Sci. 2025 Oct 29;16(48):23047–54. doi: 10.1039/d5sc05541e (PMC12584132; doi:10.1039/d5sc05541e)
Supplement: SC-016-D5SC05541E-s001 [file SC-016-D5SC05541E-s001.pdf]

Supplementary Information (SI) for Chemical Science.
This journal is © The Royal Society of Chemistry 2025

Supporting Information

Efficient and Rapid Cell Surface Functionalization: A Sub-Minute Selenol-Yne Click Reaction for Bioconjugation

Fangjian Shan, Xingyu Heng, Lihua Yao, Guichuan Xu, Jun Hu, Xiangqiang Pan*, and Gaojian Chen*

Table of Contents

Table of Contents 1

Section 1. Materials, Cell Lines and Instruments 4

Material 4

Cell Lines 4

Experimental animals 4

Instruments 5

Section 2. DFT method and Free Energy Diagram 5

Section 3. Experimental procedures 9

Small molecule model reaction 9

Reaction selectivity of monoaddition and diaddition 9

UV-Vis absorption spectra of HSeOH, AA and DSeOH 10

Kinetic monitoring of the SYC reaction 10

Kinetic monitoring of thiol-alkyne and amine-alkyne reactions 11

Reaction selectivity of SYC in the presence of amino and thiol groups 12

Cell Surface Modification with FITC-A 12

Cell viability assay 13

Loading of cellular backpacks (mesoporous silica SBA-A) 13

T-pM, T, and T-pM/pM interactions with HeLa cells 14

Preparation of Whole-Cell Tumor Vaccines 15

Detection of CD80 and CD86 Expression on DC2.4 Cells 15

Glycosylation of zebrafish V-shaped myotomes 15

Section 4. Synthesis and preparation 16

Synthesis of DSeOH 16

Synthesis of Chol-SeH 17

Synthesis of FITC-A 18

Synthesis of pMF-A and pM-A........ 18

Preparation of SBA-A 21

Section 5. Spectra 22

IR Spectrum of Chol-NH_2 22

IR Spectra of SBA-15, SBA-NH_2 and SBA-15-A 22

^{1}H NMR Spectrum of HSeOH (400 MHz, D_2O:DMF-d_7 = 7:1) 23

^{13}C NMR Spectrum of HSeOH (150 MHz, D_2O:DMF-d_7 = 7:1) 24

^{1}H NMR Spectrum of AA (400 MHz, DMSO-d_6) 25

^{13}C NMR Spectrum of AA (150 MHz, DMSO-d_6) 26

^{1}H NMR Spectrum of DSeOH (400 MHz, D_2O:DMF-d_7 = 7:1) 27

^{13}C NMR Spectrum of DSeOH (150 MHz, D_2O:DMF-d_7 = 7:1) 28

^{1}H NMR Spectrum of Chol-NH_2 (400 MHz, $CDCl_3$) 29

^{13}C NMR Spectrum of Chol-NH_2 (150 MHz, $CDCl_3$) 30

^{1}H NMR Spectrum of Chol-SeH (400 MHz, $CDCl_3$) 31

^{13}C NMR Spectrum of Chol-SeH (150 MHz, $CDCl_3$) 32

^{1}H NMR Spectrum of FITC-A (400 MHz, $CDCl_3$) 33

^{1}H NMR Spectrum of mPEG-A (400 MHz, $CDCl_3$) 34

^{1}H NMR Spectrum of MAG (400 MHz, DMSO-d_6) 35

^{1}H NMR Spectrum of MAG (300 MHz, D_2O) 36

^{1}H NMR Spectrum of pM, pM-A, pMF and pMF-A (400 MHz, DMSO-d_6) 37

HRMS Spectrum of AA 38

HRMS Spectrum of HSeOH 39

HRMS Spectrum of DSeOH 40

HRMS Spectrum of Chol-NH_2 41

HRMS Spectrum of Chol-SeH 42

References 43

Section 1. Materials, Cell Lines and Instruments

Material

Methyl propiolate, Cholesteryl chloroformate, Diethylene glycol bis(3-aminopropyl) ether, D-(+)-Glucosamine hydrochloride, Methacryloyl chloride, 3-(Triethoxysilyl)propylamine (APTES), Azobis(isobutyronitrile) (AIBN), 4-Cyano-4-[(phenylcarbonothioyl)thio]pentanoic Acid (CPADB), 4-Dimethylaminopyridine (DMAP), β-mercaptoethanol, 1-Hydroxybenzotriazole (HOBt), 1,3-Diisopropylcarbodiimide (DIC), Triethylamine (Et_3N), Potassium Carbonate (K_2CO_3), Sodium Chloride (NaCl), Potassium Chloride (KCl), Calcium Chloride ($CaCl_2$), Magnesium Sulfate ($MgSO_4$), Methylene blue, N-Phenylthiourea (PTU) were purchased from Aladdin Co., Ltd (Shanghai, China). Propiolic acid was purchased from Energy Chemical Co., Ltd. (Shanghai, China). SBA-15, *N,N*-Dimethylformamide-d_7 (DMF-d_7), Chloroform-d ($CDCl_3$) were purchased from J&K Scientific (Beijing, China). Cholesterol (Chol), PBS Buffer Premixed Tablets (pH 7.4), Fluorescein O-methacrylate were purchased from Macklin Biochemical Technology Co., Ltd., (Shanghai, China). FITC-PEG-NH_2 (FITC -NH_2) and mPEG-NH_2 was purchased from Ponsure Biotechnology (Shanghai, China). Acetone, toluene, methanol, Methylene Chloride (DCM), Dimethyl Sulfoxide (DMSO), *N,N*-Dimethylformamide (DMF) were purchased from Chinasun Specialty Products Co., Ltd. (Jiangsu, China). Roswell Park Memorial Institute (RPMI) 1640 media, fetal bovine serum (FBS), and penicillin sulfate and streptomycin (PS) were purchased from Gibco (New York, USA). DMEM medium was purchased from Biosharp (Anhui, China). Plasmocin™ was obtained from Invivogen Co., Ltd (San Diego, USA). The antimouse CD80 (PE), anti-mouse CD86 (APC) were purchased from BioLegend (San Diego, USA). Deionized water (DIW), purified to a minimum resistivity of 18.25 MΩ/cm by a Millipore water purification system, was used in all experiments.

Cell Lines

Jurkat (Clone E6-1, T cells), Mouse dendritic cells (DC2.4) and B16-OVA (Enzyme Research Biotechnology Co., ltd (Shanghai, China)) were cultured in RPMI 1640 medium. HeLa cells (Enzyme Research Biotechnology Co., ltd (Shanghai, China)) were cultured in DMEM medium with high glucose. Mediums contain 10% FBS, 100 U/mL penicillin, 100 μg/mL streptomycin and 5 μg/mL Plasmocin™ (except for Jurkat cell medium, which lacked this drug) prophylactic at 37°C in 5% CO_2. In addition, the medium of both cells was changed every two days.

Experimental animals

Zebrafish embryos of the wild-type AB strain were obtained from Shanghai FishBio Co., Ltd. (Shanghai, China). Embryos and larvae were maintained in E3 zebrafish embryo medium (5 mM NaCl, 0.17 mM KCl, 0.33 mM $CaCl_2$, 0.33 mM $MgSO_4$, 10^{-5}% methylene blue, and 0.2 mM PTU) at 28.5°C. Larvae were fed with starter feed beginning at 5 dpf. All experiments were conducted in accordance with guidelines approved by the Soochow University Committee on the Use and Care of Animals.

Instruments

NMR: ^1H and ^{13}C NMR spectra were recorded on a Bruker Avance 300 at 300/75 MHz, Bruker Avance 400 at 400/100 MHz or Agilent DD2 600MHz/150 MHz. Chemical shifts are presented in parts per million (δ) relative to solvent peak used as internal standard. Coupling constants (J) in ^1H NMR spectra are given in Hz. The resonance multiplicities are described as s (single), d (doublet), t (triplet), q (quartets) or m (multiplet). When using a D_2O/DMF-d_7 mixed solvent, the deuterium oxide (D_2O) was first prepared into a deuterated phosphate buffer using PBS tablets before use.

FT-IR: Fourier transform infrared (FT-IR) spectra were measured on a Bruker Ⅱ FT-IR spectrometer equipped with Attenuated Total Reflection (ATR) accessories.

HRMS: High-resolution mass spectrometry (HR-MS) results were obtained with a Waters Xevo G2-XS Tof spectrometer.

LSCM: Laser scanning confocal fluorescent microscope (Leica, TCS-SP5) equipped with diode laser 405 nm, multiline argon laser, 458, 476, 488, and 514 nm was utilized for capturing fluorescence cell images assisted with image analysis software.

IM: An inverted microscope (Nikon, Eclipse Ti-S) equipped with a video camera (Basler, acA 1600-20um) was used to capture particle-cell and cell-cell interactions. An inverted microscope (Olympus, IX71) equipped with a video camera was used for fluorescence observation and imaging of zebrafish.

FCM: The fluorescence intensities of cells were obtained by flow cytometry (FCM) by a FACSVerse flow cytometer (BD Biosciences, Franklin Lakes, NJ, USA).

SEM: A scanning electron microscope (Regulus SU8100, Hitachi) was used to capture SEM images.

SEC: Both the number-average molecular weight ($M_{n,\ GPC}$) and molecular weight distribution ($Đ$) of all the polymers were determined by Waters 1515 size exclusion chromatography (SEC) equipped with a refractive-index detector, using UltrahydrogelTM 6×40 mm Guard Colum at 35°C. A buffer containing 0.2 M $NaNO_3$ and 0.01 M NaH_2PO_4, adjusted to pH 9.0 using NaOH, was used as the eluent at a flow rate of 1 mL/min. The molar masses were calibrated with narrow polydispersity using Polyethylene glycol standards.

Microinjection system: Microinjection was performed using a pressure microinjector (Warner Instruments, PLI-10) in conjunction with a stereomicroscope (Optech, Serie GZ 808). The injection needles were pulled from capillary glass tubes (WPI, TW120F-4).

Section 2. DFT method and Free Energy Diagram

Geometry optimizations were optimized using a of three-parameter Becke-style hybrid functional (m062x) with the Pople basis set 6-311+G(d,p). Frequency analysis was conducted

at the same level of theory to obtain zero-point energy (ZPE) and thermal energy corrections at 298.15 K. The effects of solvation (water) were included using the CPCM implicit solvent model. All DFT calculations were carried out with the GAUSSIAN 09 package1. The Optimized Cartesian Coordinates and Free Energy Diagram are listed below.

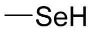

C	-5.06280	-0.13862	0.00981
Se	-3.36451	0.85139	-0.15486
H	-4.86064	-1.11613	0.43372
H	-5.46002	-0.24072	-0.99499
H	-5.75527	0.42550	0.62369
H	-3.54151	1.69133	1.03872

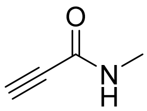

C	7.62275	-6.58724	-1.06815
C	8.47048	-5.74367	-0.95879
C	9.52209	-4.76962	-0.66182
N	9.38411	-3.57695	-1.25440
C	10.36630	-2.51743	-1.11890
O	10.43410	-5.06455	0.11341
H	8.60591	-3.44302	-1.88012
H	10.03960	-1.76919	-0.39645
H	10.51560	-2.04141	-2.08362
H	11.30290	-2.95024	-0.77882
H	6.87115	-7.33413	-1.18987

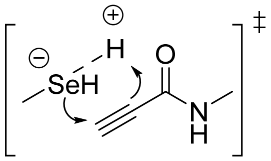

C	-12.93700	-0.14470	1.67384
C	-11.66110	0.24049	1.69029
C	-10.93400	0.58652	0.46093
N	-10.21310	-0.39740	-0.13176
C	-9.15375	-0.11547	-1.09311
O	-10.88750	1.75721	0.03375
H	-10.20370	-1.29501	0.32860
H	-9.55716	0.27830	-2.02748
H	-8.62440	-1.04796	-1.29318

H	-8.44648	0.61220	-0.68417
Se	-13.49600	-0.42119	3.52406
C	-14.22600	1.32384	4.03675
H	-11.95550	0.01266	3.44486
H	-14.29240	1.32998	5.12008
H	-15.21440	1.40812	3.59236
H	-13.55180	2.08989	3.65992
H	-13.65700	-0.32821	0.88428

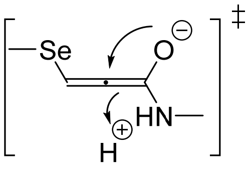

C	-2.50636	0.22017	-2.56991
C	-1.30968	0.07110	-1.91339
C	-1.26080	0.15977	-0.44785
N	-0.91601	1.32539	0.14188
C	-0.40260	1.30507	1.51786
O	-1.44765	-0.89216	0.17877
H	-0.49460	2.01344	-0.47430
H	-1.07428	0.72757	2.15169
H	-0.33609	2.32490	1.89210
H	0.59653	0.84994	1.55338
Se	-4.29730	0.36387	-1.77121
C	-5.30067	0.22455	-3.47281
H	-1.81960	1.23136	-2.52547
H	-4.76149	-0.45197	-4.13485
H	-5.41151	1.20549	-3.93060
H	-6.27449	-0.20081	-3.23390
H	-2.56271	0.03809	-3.64586

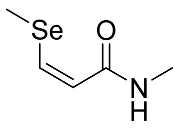

C	7.43547	-5.58128	-1.89538
C	8.40310	-5.75372	-0.99430
C	9.39144	-4.70699	-0.69641
N	10.26930	-5.00090	0.27777
C	11.38720	-4.13499	0.59798
O	9.41260	-3.63038	-1.31109
H	10.23120	-5.91961	0.69018

H	12.20240	-4.25037	-0.11742
H	11.73920	-4.37845	1.59684
H	11.04750	-3.10157	0.58560
Se	7.06900	-3.97049	-2.82020
C	5.32744	-4.60485	-3.41930
H	8.49221	-6.70284	-0.48064
H	4.69735	-4.78034	-2.55267
H	5.44286	-5.51172	-4.00421
H	4.89057	-3.81953	-4.03307
H	6.72990	-6.37399	-2.10813

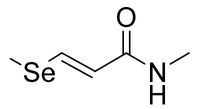

C	7.16827	-10.88560	1.45552
C	8.16585	-10.77890	0.58336
C	8.94617	-9.51835	0.56361
N	10.01660	-9.51910	-0.24211
C	10.94530	-8.41102	-0.28669
O	8.63341	-8.53091	1.24203
H	10.23040	-10.35090	-0.76642
H	10.43760	-7.49832	-0.60183
H	11.73830	-8.64463	-0.98911
H	11.37590	-8.23212	0.69728
Se	6.11664	-12.44060	1.70812
C	5.76661	-12.01230	3.58230
H	8.47849	-11.59700	-0.05257
H	6.70851	-11.95760	4.11931
H	5.14522	-12.80000	3.99853
H	5.23605	-11.06600	3.63049
H	6.92630	-10.05370	2.10796

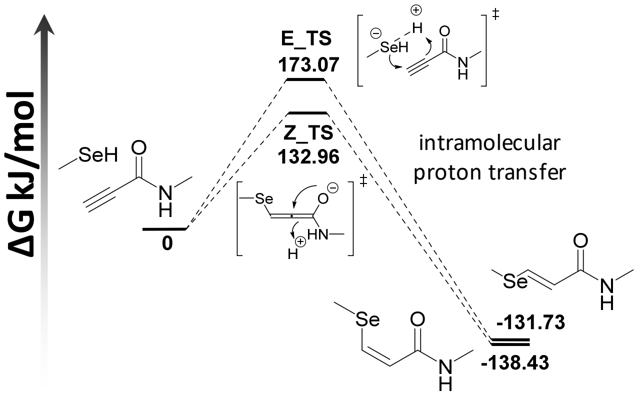

Figure S1. Calculated free energy diagram for the reaction between selenol and alkyne.

Section 3. Experimental procedures

Small molecule model reaction

A solution of γ-selenobutyrolactone (4.0 μL, 0.045 mmol) in DMF-d_7 (100 μL) was prepared in a glovebox, followed by the addition of ethylenediamine (2.7 μL, 0.045 mmol). The mixture was stirred at room temperature for 12 hours, then transferred to an NMR tube. Afterward, AA (3.7 mg, 0.022 mmol) in PBS (700 μL, prepared with deuterium oxide) was added, and the ^{1}H NMR spectrum was recorded immediately.

Reaction selectivity of monoaddition and diaddition

HSeOH (64.70 μL, 0.45 mmol，4.8 eq) in DMF (143 μL) was mixed with AA (15.53 mg, 0.095 mmol, 1 eq) in PBS (1 mL). The mixture was stirred in the glove box for 1 hour. Then, the mixture was lyophilized, yielding a pale yellow solid. The dried product was dissolved in 600 μL deuterated solvent (D_2O:DMF-d_7 = 7:1) for ^{1}H NMR analysis ; the corresponding NMR spectrum is shown below.

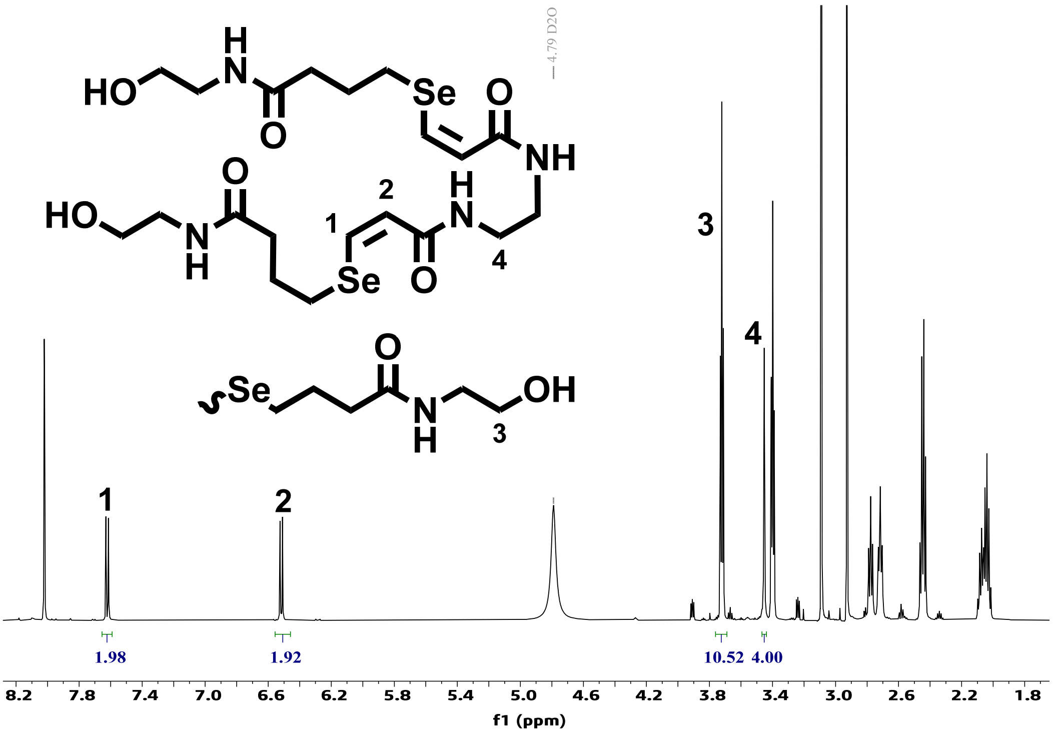

Figure S2 Reaction results with excess HSeOH, n_{HSeOH}: n_{AA} = 4.8:1.

^{1}H NMR (400 MHz, D_2O:DMF-d_7 = 7:1).

UV-Vis absorption spectra of HSeOH, AA and DSeOH

AA and DSeOH solutions (3.05 μmol/mL) and an HSeOH solution (6.08 μmol/mL) were prepared in PBS. UV-Vis absorption spectra were recorded in the 275-450 nm range. AA shows an absorption peak at 329 nm, while HSeOH does not absorb at this wavelength (Figure S3A). Therefore, 329 nm was chosen as the monitoring wavelength for kinetic studies.

Kinetic monitoring of the SYC reaction

AA solutions at concentrations of 1.50, 3.06, 4.57, and 6.08 μmol/mL (2 mL each) and HSeOH solutions at 6.00, 12.24, 18.28, and 24.32 μmol/mL (1 mL each) were prepared using PBS. AA solutions were added to quartz cuvettes, then mixed with the corresponding HSeOH solutions under light-protected conditions. Absorbance at 329 nm was recorded at 0.4-second intervals. To establish a standard curve, absorbance measurements of AA and DSeOH at different concentrations were taken under the same instrument parameters. Since the absorbance is no longer linearly correlated with the concentration when the DSeOH concentration is high, the experiment controls the DSeOH concentration below 4.05 μmol/mL. The solute concentration and absorbance follow the relationship below:

$$[AA] + [DSeOH] = [AA]_0$$

$$A_{sum} = A_{AA} + A_{HSeOH} + A_{DSeOH}$$

$$A_{AA} = k_{AA}[AA] + b_{AA}$$

$$A_{DSeOH} = k_{DSeOH}[DSeOH] + b_{DSeOH}$$

Let $[AA]$ and $[DSeOH]$ represent the concentrations of AA and DSeOH, and $[AA]_0$ is the initial concentration of AA at Time = 0. A_{sum} is the total absorbance, A_{AA} the absorbance of AA, and A_{DSeOH} the absorbance of DSeOH. The absorption contribution of HSeOH at 329 nm can be ignored. Based on the above equation and the obtained standard curve (Figure S3B and C), the following relationship between conversion rate and A_{sum} is obtained:

$$Conversion\ (\%) = \frac{[AA]_0 - [AA]}{[AA]_0} = \frac{A_{sum} - 0.036 - 0.095[AA]_0}{0.775[AA]_0} \times 100\%$$

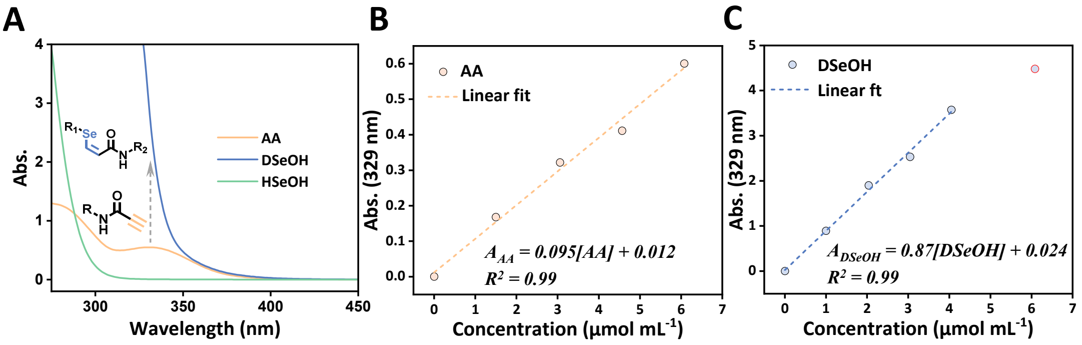

Figure S3. (A) UV absorption spectra of AA, HSeOH, and DSeOH; (B and C) Concentration standard curves of AA and DSeOH.

Kinetic monitoring of thiol-alkyne and amine-alkyne reactions

To investigate the reaction rates of amino and thiol groups with alkynes under physiological conditions, β-mercaptoethanol and ethanolamine were used as model molecules (Figure S4A). To prevent ethanolamine from altering the pH of the reaction system, the pH was pre-adjusted to 7.4 using concentrated hydrochloric acid (37%). At this point, the theoretical concentration of ethanolamine hydrochloride was 6.95 mmol/mL. To ensure an excess of amino groups, 5.84 μL of the ethanolamine hydrochloride solution (0.041 mmol, 2.4 eq, calculated based on amino groups) was added to 75 μL of DMF-d_7 and set aside. Similarly, β-mercaptoethanol (2.86 μL, 0.041 mmol, 2.4 eq) was added to DMF-d_7 and set aside. Afterward, AA (2.78 mg, 0.017 mmol) in PBS (525 μL, prepared with deuterium oxide) was added, and the ^{1}H NMR spectrum was recorded every 10 minutes.

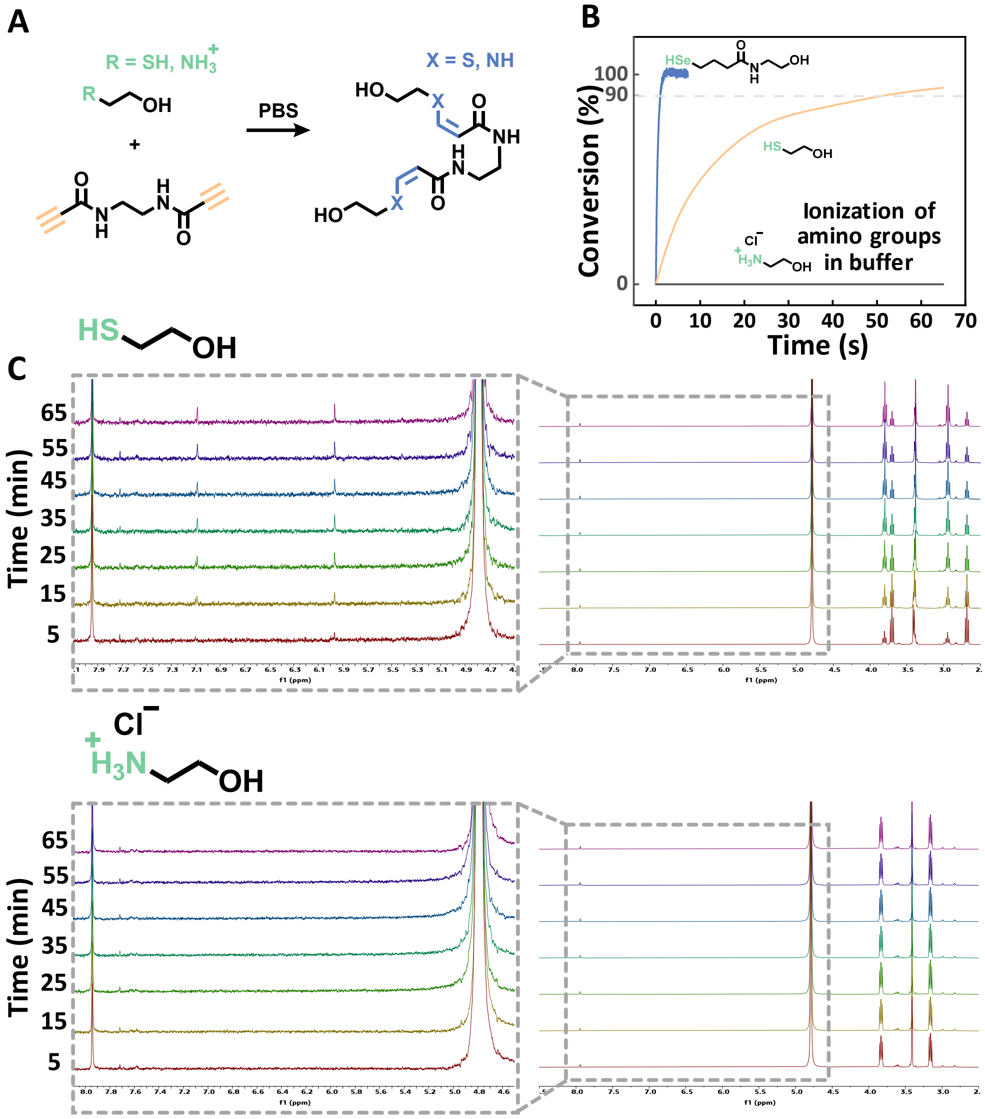

Figure S4. (A) Model reactions between thiol/amino groups and alkyne in PBS at room temperature; (B) The reaction conversion–time profile and (C) the corresponding ^{1}H NMR spectra at various time points. The conversion of the selenol species was determined using the UV-Vis method described in "Kinetic monitoring of the SYC reaction".

Reaction selectivity of SYC in the presence of amino and thiol groups

AA (7.8 mg, 0.048 mmol, 1 eq) was dissolved in 1 mL PBS to prepare the AA solution. Ethanolamine (8.62 μL, 0.144 mmol, 3 eq) was dissolved in 0.5 mL PBS, and the pH was adjusted to 7.4 using 10 M HCl. In a glove box, HSeOH (13.90 μL, 0.096 mmol, 2 eq) and β-mercaptoethanol (8.62 μL, 0.144 mmol, 3 eq) were dissolved in 214.5 μL DMF. The solutions were combined, stirred for 1 hour, and lyophilized. The dried product was dissolved in 600 μL deuterated solvent (D_2O:DMF-d_7 = 7:1) for ^{1}H NMR analysis ; the corresponding NMR spectrum is shown below.

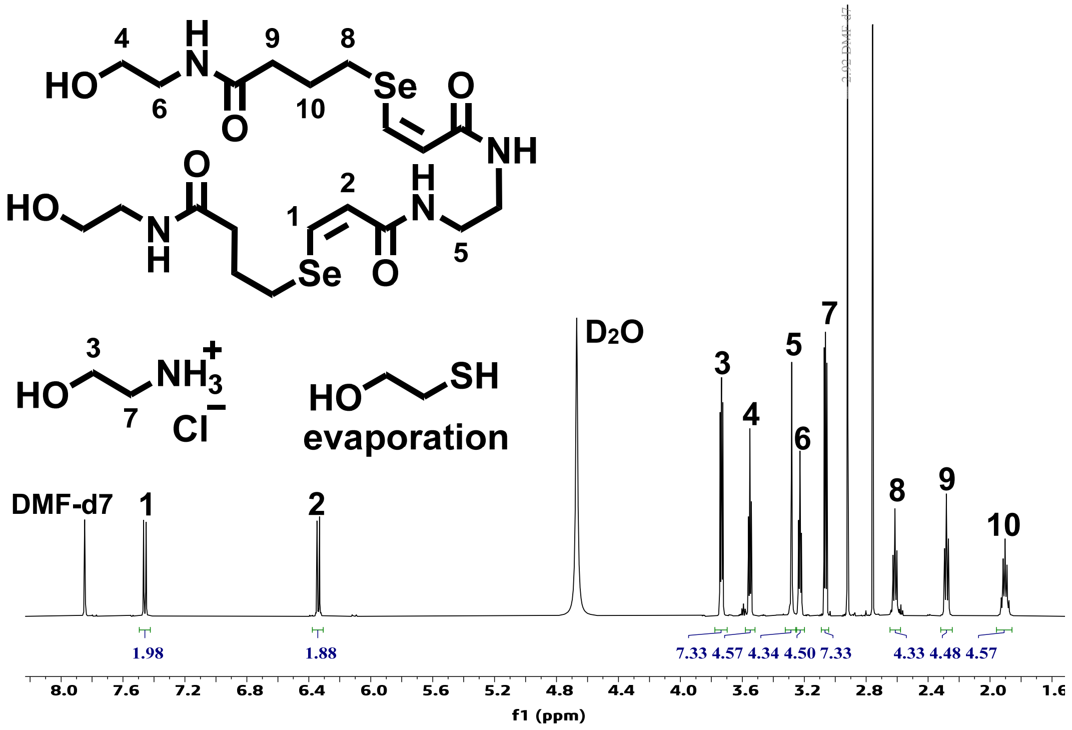

Figure S5 Experimental results on the reaction selectivity of SYC in the presence of amino and thiol groups. ^{1}H NMR (400 MHz, D_2O:DMF-d_7 = 7:1).

Cell Surface Modification with FITC-A

90,000 HeLa cells were seeded into a confocal dish and cultured for 24 hours to allow full attachment. The medium was then replaced with 1 mL DMEM, and the cells were pre-warmed in a 37°C incubator for 30 minutes. After pre-warming, 5 μL of Chol-SeH solution (2.6 mM in DMF) or Chol solution (2.6 mM in DMF) was added and mixed thoroughly, followed by incubation at 37°C for 10 minutes. The medium was discarded, and the cells were washed twice with PBS. Then, 200 μL of FITC-A or FITC-NH_2 solution (125 μg/mL in PBS) was added and incubated at room temperature for 15 minutes. After incubation, the solution was discarded, the cells were washed twice with PBS, and imaging was performed using a confocal microscope.

Cell viability assay

Hela, B16-OVA, and Jurkat cells were seeded in 6-well plates at a density of 90,000 cells per well and cultured for 24 hours before subsequent experiments. Cell surface modification with mPEG-A (at a molar concentration equivalent to that of FITC-A) was performed on Hela, B16-OVA and Jurkat cells following the same protocol as used for 'Cell Surface Modification with FITC-A'. Since Jurkat cells are suspension cells, the supernatant was removed by centrifugation. After surface modification, Hela, B16-OVA, and Jurkat cells were collected and reseeded into 96-well plates at 10,000 cells per well. Untreated cells were used as a control. After 24 hours of incubation, cell viability was assessed using the Cell Counting Kit-8 (CCK-8), following a previously reported protocol[1].

Loading of cellular backpacks (mesoporous silica SBA-A)

Cell image tracking: 18,000 HeLa cells were seeded per well in a 48-well plate and cultured for 24 hours. The medium was then replaced with 250 µL of DMEM, and the plate was pre-warmed in a 37°C incubator for 30 minutes. After pre-warming, 12 µL of Chol-SeH solution (2.6 mM in DMF) or Chol solution (2.6 mM in DMF) was added and mixed thoroughly, followed by incubation at 37°C for 10 minutes. The medium was discarded, and the cells were washed twice with PBS. Then, 1 mL of SBA-A or SBA-15 suspension (165 particles/mL in PBS) was added. Then, time-lapse imaging was immediately performed using an optical microscope.

SEM imaging: A 5×5 mm square silicon chip was placed in each well of a 48-well plate. 18,000 HeLa cells were seeded per well and cultured for 24 hours. The medium was then replaced with 250 µL of DMEM, and the plate was pre-warmed in a 37°C incubator for 30 minutes. After pre-warming, 12 µL of Chol-SeH solution (2.6 mM in DMF) or Chol solution (2.6 mM in DMF) was added and mixed thoroughly, followed by incubation at 37°C for 10 minutes. The medium was discarded, and the cells were washed twice with PBS. Then, 1 mL of SBA-A or SBA-15 suspension (165 particles/mL in PBS) was added. After incubation at room temperature for 15 minutes, the cells were fixed with 4% paraformaldehyde for 15 minutes. This was followed by dehydration through a graded ethanol series: 50%, 70%, 80%, 95%, and 100%, with each step lasting 10 minutes. The surface of the silicon wafer sample with cells was sputter-coated with platinum using a sputter coater, followed by SEM imaging.

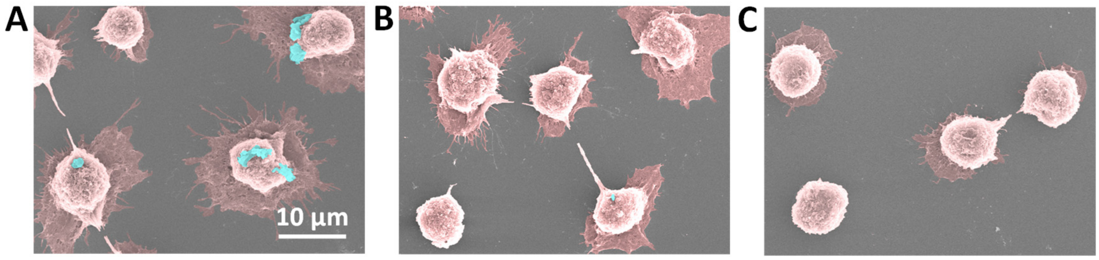

Figure S6. SEM images of cells treated with (A) Chol-SeH and BSA-A, (B) Chol and BSA-A, (C) Chol-SeH and SBA, red indicates cells and blue indicates SBA particles.

Drug delivery: 25 µL of Nile Red solution (1 mg/mL in DCM) was added to 3 mg of SBA-A. The particles were washed with ethanol until the wash solution became colorless to obtain SBA-A-Nile red, simulating drug molecule loading onto SBA-A. 100 µL of Jurkat (1.2 million cells/mL) was mixed with 14 µL of Chol-SeH solution (1 mg/mL in DMF); The control group used Chol solution (2.6 mM in DMF). Then, 30 µL of SBA-A-Nile Red suspension (3 mg/mL) was added. After 15 minutes of co-incubation, 30 µL of the mixture was transferred into a 48-well plate containing 1 mL of PBS and observed using fluorescence microscopy.

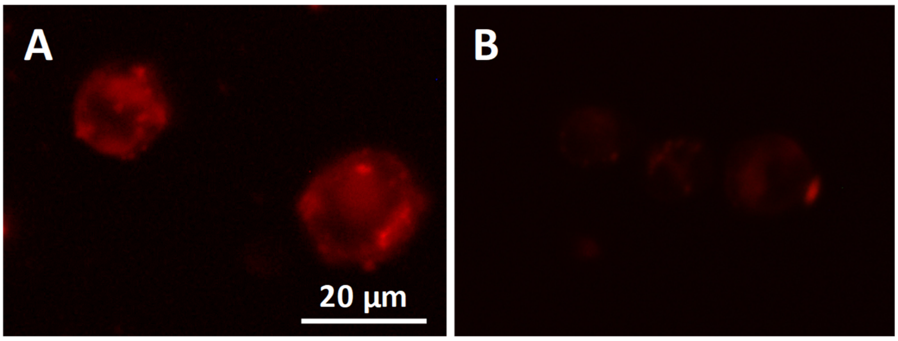

Figure S7. Fluorescence microscopic images of cells treated with (A) Chol-SeH and BSA-A-Nile red, (B) Chol and SBA-A-Nile red.

T-pM, T, and T-pM/pM interactions with HeLa cells

HeLa cells were pre-seeded in a 48-well plate at 40,000 cells per well in 0.6 mL of medium per well. After 12 hours of incubation to allow cell attachment, the medium was removed, and subsequent experiments were carried out.

T-pM group：Jurkat cells (1 million cells in 3 mL medium) were treated with 15 µL of Chol-SeH solution (1 mg/mL in DMF) and incubated at 37°C for 10 minutes. The medium was then removed, and 0.6 mL of pM-A solution (1 mg/mL in PBS) was added. After incubation at room temperature for 15 minutes, the PBS was removed and replaced with 0.6 mL of fresh culture medium to obtain T-pM cells. 200,000 T-pM cells were then added to each well of a 48-well plate containing pre-seeded HeLa cells, and the medium volume was adjusted to 0.6 mL per well. Time-lapse imaging was then performed using an optical microscope.

In order to verify the successful modification of glycopolymers on the cell surface, pM-A was replaced with pMF-A while keeping other conditions unchanged to prepare T-pMF.

T group: Untreated Jurkat cells (200,000 cells in 0.6 mL medium) were added to wells of a 48-well plate containing pre-seeded HeLa cells. Time-lapse imaging was then performed using an optical microscope to track cell interactions.

T-pM/pM group：All other conditions were the same as in the T-pM group, except that during the interaction between T-pM and HeLa cells, the culture medium contained 2 mg/mL of pM.

Preparation of Whole-Cell Tumor Vaccines

B16-OVA cells (2,000,000 cells in 1 mL) were placed in a 35 mm culture dish and inactivated by UV irradiation (253 nm, 21 μW/cm²) for 30 minutes. The inactivated B16-OVA cells (500,000 cells in 3 mL medium) were then treated with 15 μL of Chol-SeH solution (1 mg/mL in DMF) or Chol solution (1 mg/mL in DMF) and incubated at 37°C for 10 minutes. After removing the medium, 0.6 mL of pG-A solution (1 mg/mL in PBS) or PBS was added. Following 15 minutes of incubation at room temperature, the PBS was removed and replaced with 1 mL of fresh culture medium to obtain the whole-cell tumor vaccines: B16O-pM, B16O-SeH, B16O/pM or B16O.

In order to verify the successful modification of glycopolymers on the cell surface, pM-A was replaced with pMF-A while keeping other conditions unchanged to prepare B16O-pMF.

Detection of CD80 and CD86 Expression on DC2.4 Cells

DC2.4 cells were retrieved through enzymatic digestion from T25 flask and seeded in six-well plates at a density of 100,000/well. The plates were placed in the incubator overnight to stabilize the cell state. Then, the supernatant in the plates was removed and the WTCV was added (200,000/well) to the well plate which was incubated for 48 hours. Subsequently, removing the supernatant of each well, the cells were retrieved through enzymatic digestion and collected into the centrifuge tubes, into which a certain amount of fluorescent antibodies (CD80 (PE) (0.5 μL/test) and CD86 (APC) (0.25 μL/test)) were added. After incubation for 30 minutes, the supernatant of the centrifuge tube was removed by centrifugation and the cells in the tube were washed twice with PBS. Finally, the buffer (sterile PBS containing 1% FBS) was added to the centrifuge tube, mixed, and transferred to the flow tubes for detection.

Glycosylation of zebrafish V-shaped myotomes

Preparation of reagents: Add 5 μL of Chol or Chol-SeH (1 mg/mL in MDF) to 1 mL of PBS and mix thoroughly for use. Prepare injection solutions of pMF or pMF-A in PBS at a concentration of 125 μg/mL.

Microinjection apparatus setup: Load 2 μL of the prepared solution into a capillary glass needle using a pipette. Secure the reagent-loaded glass needle onto the holder of the microinjection apparatus. Under a stereomicroscope, break off the needle tip with forceps until the liquid can flow through. Adjust the microinjection system to deliver an injection volume of 2 nL per pulse.

After zebrafish larvae (7 dpf) were anesthetized at low temperature (8 °C), Chol or Chol-SeH was injected into the V-shaped myotomes, followed by the injection of pMF or pMF-A into the common cardinal vein. The injected larvae were subsequently incubated at 28.5 °C for 1 hour. Fluorescence imaging and photography were performed using a fluorescence inverted microscope.

Section 4. Synthesis and preparation

Synthesis of DSeOH

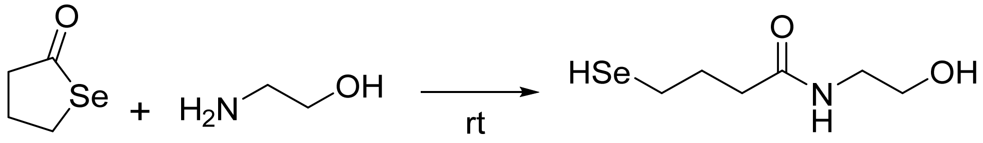

HSeOH was synthesized via the ethanolamine-induced ring-opening of γ-selenobutyrolactone. Ethanolamine (340 μL, 5.5 mmol) and γ-selenobutyrolactone (500 μL, 5.5 mmol) were mixed and stirred at room temperature for 12 hours in a glovebox, yielding a clear, viscous liquid. IR (ATR): ν_{max} = 3431, 3284, 3086, 2934, 2870, 1634, 1543. ^{1}H NMR (400 MHz, D_2O:DMF-d_7 = 7:1) δ 3.65 (t, *J* = 5.6 Hz, 2H), 3.32 (t, *J* = 5.6 Hz, 2H), 2.56 (t, *J* = 7.2 Hz, 2H), 2.37 (t, *J* = 7.4 Hz, 2H), 1.96 (p, *J* = 7.3 Hz, 2H). ^{13}C NMR (150 MHz, D_2O:DMF-d_7 = 7:1): δ 174.89 (C=O, C-1), 58.58 (CH_2, C-2), 40.04 (CH_2, C-3), 34.59 (CH_2, C-4), 29.17 (CH_2, C-5), 13.19 (CH_2, C-6). HRMS (ESI, m/z): $[M+H]^+$ calcd for $C_6H_{14}NO_2Se^+$ 212.0190 found 212.0187.

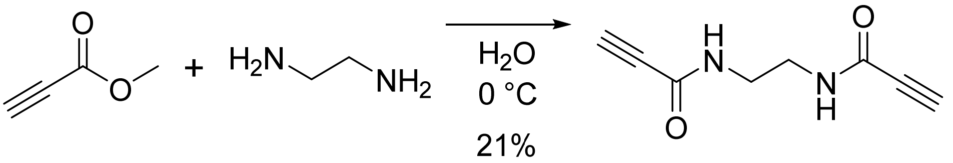

AA was synthesized following previously reported procedures with slight modifications. Briefly, a solution of ethylenediamine (3.11 mL, 23.2 mmol) in water (20 mL) was prepared and maintained at 0°C under constant stirring. Methyl propiolate (4.23 mL, 47.6 mmol) was then added dropwise over 1 hour using a syringe pump. The reaction mixture was stirred for an additional 6 hours at 0°C, during which a yellow suspension with pale yellow solids was formed. The resulting mixture was extracted with ethyl acetate (20 mL × 3). After evaporation of the solvent under reduced pressure, the crude product was purified by recrystallization ($CHCl_3$:MeOH = 5:1) to afford AA as a pale-yellow solid. IR (ATR): ν_{max} = 3293, 3248, 3067, 2109, 1631, 1552. ^{1}H NMR (400 MHz, DMSO-*d*$_6$) δ 8.74 (s, 2H), 4.13 (s, 2H), 3.20 – 3.11 (m, 4H). ^{13}C NMR (150 MHz, DMSO-d_6): δ 151.88 (C=O, C-1), 78.27 (C≡C, C-2), 75.76 (C≡C, C-3), 38.20 (CH_2, C-4). HRMS (ESI, m/z): $[M+H]^+$ calcd for $C_8H_9N_2O_2^+$ 165.0664 found 165.0665.

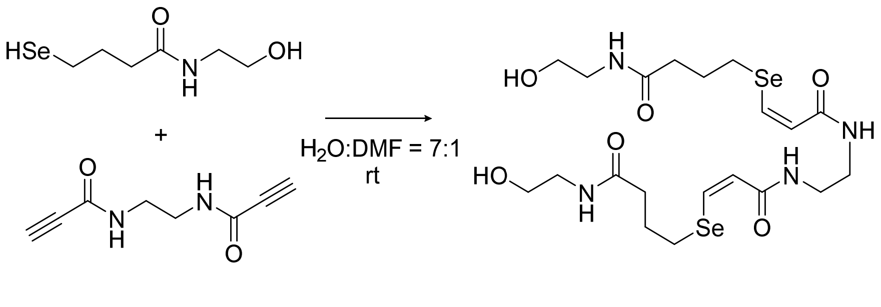

DSeOH was obtained through the Selenol-Yne Click Reaction. HSeOH (121.0 mg, 0.576 mmol)

in DMF (429 μL) was mixed with AA (46.6 mg, 0.288 mmol) in water (3 mL). The mixture was dried in a vacuum oven, yielding a white solid. IR (ATR): v_{max} = 3433, 3259, 3073, 2936, 1646, 1632, 1570, 1543. ^{1}H NMR (400 MHz, D_2O:DMF-d_7 = 7:1) δ 7.53 (d, *J* = 9.6 Hz, 2H), 6.40 (d, *J* = 9.6 Hz, 2H), 3.65 (t, *J* = 5.6 Hz, 4H), 3.38 (d, *J* = 3.9 Hz, 4H), 3.32 (t, *J* = 5.6 Hz, 4H), 2.72 (t, *J* = 7.3 Hz, 4H), 2.38 (t, *J* = 7.3 Hz, 4H), 2.01 (p, *J* = 7.3 Hz, 4H). ^{13}C NMR (150 MHz, D_2O:DMF-d_7 = 7:1): δ 174.43 (C=O, C-1), 167.47 (C=O, C-2), 142.30 (C=C, C-3), 117.63 (C=C, C-4), 58.51 (CH_2, C-5), 40.00 (CH_2, C-6), 37.17 (CH_2, C-7), 34.09 (CH_2, C-8), 25.85 (CH_2, C-9), 25.25 (CH_2, C-10). HRMS (ESI, m/z): $[M+H]^+$ calcd for $C_{20}H_{35}N_4O_6Se_2^+$ 587.0882 found 587.0884.

Synthesis of Chol-SeH

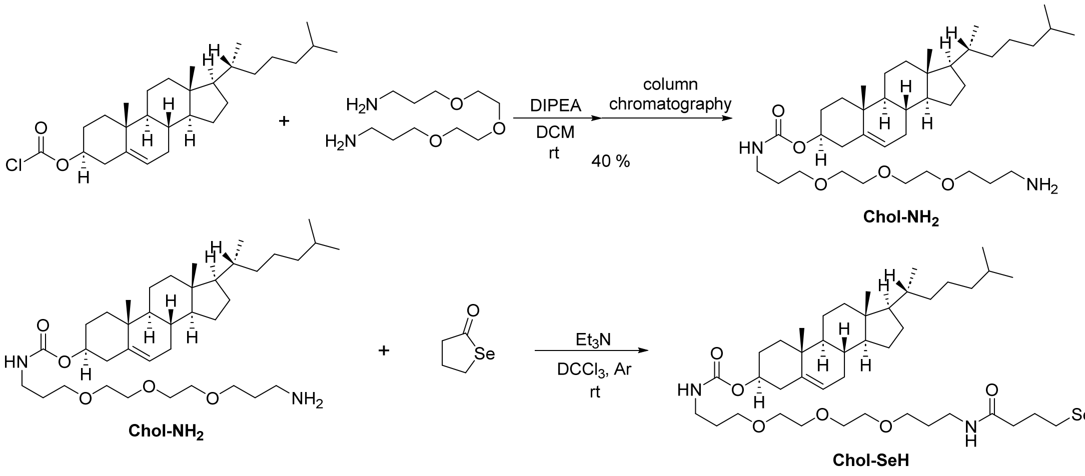

Chol-NH₂ was synthesized following previously reported procedures. 4,7,10-trioxa-1,13-tridecanediamine (1.1 mL, 5 mmol) was mixed with DIPEA (871 μL, 5 mmol) in DCM (6 mL). After 30 min, cholesteryl chloroformate (449.1 mg, 1 mmol) in DCM (4 mL) was added dropwise in the mixture. The solution was stirred overnight at rt. After removing the solvent by reduced pressure, the crude was precipitated by adding MeCN (30 mL) followed by filtration. A white sticky solid was obtained that was subsequently washed several times with distilled H_2O and air-dried. The residue was purified by flash column chromatography (silica gel, DCM:MeOH = 8:1). After rotary evaporation to remove the solvent, the product was collected with water and lyophilized to yield a white sticky solid (245 mg, 40% yield). *R*f = 0.57 (silica, DCM : MeOH = 8:1). IR (ATR): v_{max} = 3351, 2934, 2867, 1696. ^{1}H NMR (400 MHz, $CDCl_3$) δ 8.13 (s, 3H), 5.45 – 5.23 (m, 2H), 4.43 (m, 1H), 3.89 – 3.50 (m, 12H), 3.22 (m, 4H), 2.46 – 0.55 (m, 47H). ^{13}C NMR (150 MHz, $CDCl_3$): δ 156.68 (C=O, C-1), 140.00 (C=C, C-2), 122.54 (C=C, C-3), 74.41, 70.48-69.25 (CH_2OCH_2, C-4), 56.80, 56.25, 50.11, 42.42, 39.85, 39.62, 39.58, 38.72, 38.63, 37.11, 36.68, 36.29, 35.91, 32.02, 31.98, 28.34, 28.31, 28.12, 26.58, 24.40, 23.95, 22.93, 22.67, 21.15, 19.46, 18.83, 11.97. HRMS (ESI, m/z): $[M+H]^+$ calcd for $C_{38}H_{69}N_2O_5^+$ 633.5201 found 633.5205. It should be noted that the amino groups in the sample are ionized due to the influence of carbon dioxide in the air.

Chol-SeH was synthesized similarly to the method described above, through the aminolysis of γ-selenobutyrolactone. Triethylamine (2.22 μL, 0.016 mmol) and γ-selenobutyrolactone (1.4 μL, 0.016 mmol) was added to Chol-NH₂ (10 mg, 0.016 mmol) in 0.12 mL $CDCl_3$, the mixture was

stirred at room temperature for 12 hours in a glove box. After reaction $CDCl_3$ (0.6 mL) was added to mixture for NMR and Mass spectrometry test. The ring-opening reaction between Chol-NH_2 and γ-selenobutyrolactone was confirmed by ^1H and ^{13}C NMR analysis (see Section 4). ^{13}C NMR (150 MHz, $CDCl_3$): δ 172.04 (C=O, C-1), 156.24 (C=O, C-2), 139.82 (C=C, C-3), 122.36 (C=C, C-4), 74.03, 70.44-69.43 (CH_2OCH_2, C-5), 56.62, 56.06, 49.95, 42.24, 39.66, 39.44, 38.77, 38.57, 37.72, 36.95, 36.50, 36.11, 35.89, 35.71 (CH_2, C-6), 31.84, 31.80, 29.73, 29.54, 28.95 (CH_2, C-7), 28.16, 27.93, 26.54, 24.22, 23.75, 22.77, 22.51, 20.97, 19.29, 18.66, 17.10 (CH_2, C-8), 11.80. HRMS (ASAP, m/z): $[M+H]^+$ calcd for $C_{42}H_{75}N_2O_6Se^+$ 783.4785 found 783.4781.

Synthesis of FITC-A

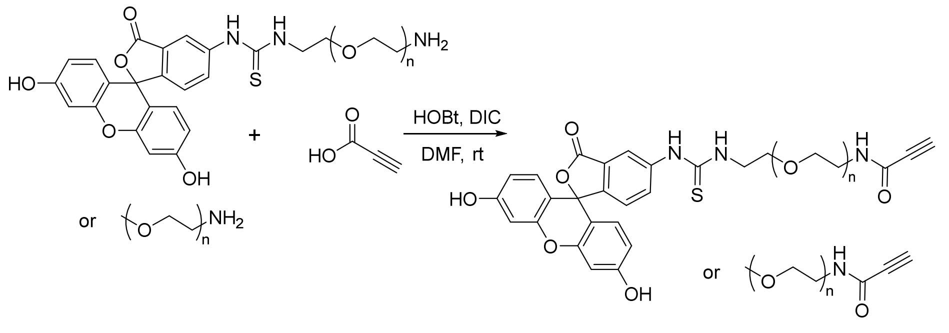

FITC-A or mPEG-A was synthesized via the condensation of FITC-PEG-NH_2 or mPEG-NH_2 with propiolic acid. 1-Hydroxybenzotriazole (1.62 mg, 12 μmol), DIC (1.88 μL, 12 μmol), and propiolic acid (0.74 μL, 12 μmol) were added to DMF (100 μL), and the mixture was stirred at room temperature for 5 minutes. FITC-PEG-NH_2 (15 mg, 3 μmol) or mPEG-NH_2 (7.5 mg, 3 μmol) was then added, and the reaction was carried out at room temperature for 4 hours. The resulting mixture was dialyzed against deionized water for 3 days and then lyophilized to afford fluffy solid product FITC-A or mPEG-A. The ^1H NMR spectrum of FITC-A and mPEG-A in Section 4 confirms the successful alkynylation of FITC-NH_2 or mPEG-NH_2 by the alkynyl hydrogen signal at 2.8 ppm.

Synthesis of pMF-A and pM-A

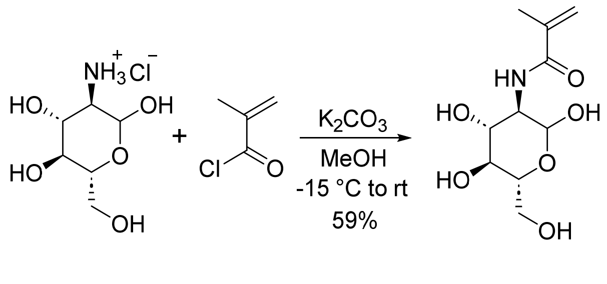

MAG was synthesized as previously reported[2, 3]. D-(+)-glucosamine hydrochloride (5 g, 20.24 mmol) and potassium carbonate (3.2 g, 23.16 mmol) were dissolved in 120 mL anhydrous methanol in a single neck round bottom flask using ice and ethanol to bathe. When the temperature of the solution drops to -15°C, methacryloyl chloride (1.8 mL, 18.53 mmol) was added dropwise. After the dripping was completed, the reaction solution was kept at -15°C for

30 minutes. The reaction proceeded for 3 hours, after which the solid in the flask was removed by filtration, and the products were purified by column chromatography (MeOH:DCM, 1:4). Finally, 3.44 g of white solid was obtained. ^{1}H NMR (400 MHz, DMSO-d_6) (α and β anomers) δ 7.62 (d, J = 8.5 Hz, 0.23H), 7.33 (d, J = 7.0 Hz, 0.75H), 6.46 (d, J = 6.4 Hz, 0.24H), 6.40 (d, J = 4.0 Hz, 0.77H), 5.74 – 5.69 (m, 0.73H), 5.68 – 5.64 (m, 0.23H), 5.37 – 5.32 (m, 0.72H), 5.32-5.29 (m, 0.25H), 4.98 (dd, J = 4.6, 2.7 Hz, 0.74H), 4.93 (m, 1H), 4.80 (d, J = 5.2 Hz, 0.24H), 4.66 (d, J = 5.0 Hz, 0.74H), 4.54 (dd, J = 7.0, 5.3 Hz, 0.23H), 4.51 (t, J = 4.9 Hz, 0.25H), 4.42 (t, J = 5.8 Hz, 0.8H), 3.74 – 3.35 (m, 5.23H), 3.21 – 2.98 (m, 1.32H), 1.86 (m, 3H). ^{1}H NMR (300 MHz, D_2O) (α and β anomers): δ (ppm) 5.62 (s, 1H), 5.50 (s, 1H), 5.25 (d, J = 3.5 Hz, 0.52H), 4.82 (s, 0.48H), 4.02 – 3.43 (m, 6H), 1.86 (s, 3H).

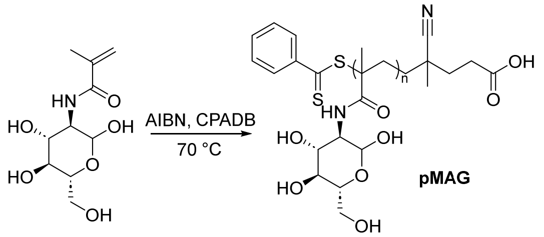

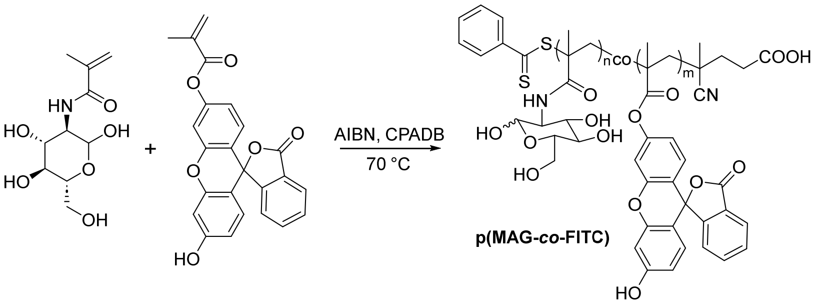

PMAG (pM) or **p(MAG-co-FITC)** was synthesized via RAFT polymerization. MAG (200 mg, 0.81 mmol), Fluorescein O-methacrylate (21 mg, 53.1 nmol), CPADB (15 mg, 53.1 nmol), and AIBN (0.87 mg, 5.31 nmol) were dissolved in 0.6 mL of DMSO in a 2 mL ampule containing a magnetic stirring bar. When synthesizing pMAG, Fluorescein O-methacrylate was not added. The solution was purged with argon for 20 minutes to remove oxygen. The ampule was then flame-sealed under an argon atmosphere and stirred at 70°C for 12 hours. After the reaction. After the reaction, the mixture was precipitated using isopropanol and then dried at room temperature in a vacuum drying oven to obtain pMAG (a pink solid) or p(MAG-co-FITC) (a bright yellow solid).

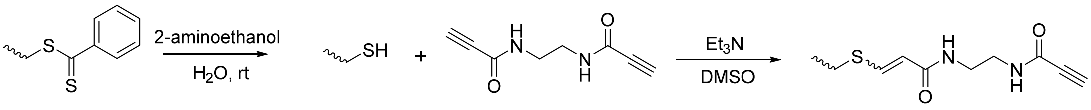

pMAG or p(MAG-*co*-FITC) — **pMAG-SH or p(MAG-*co*-FITC)-SH** — **pMAG-A or p(MAG-*co*-FITC)-A**

PMAG-SH or **p(MAG-co-FITC)-SH** was prepared through the alkaline cleavage of the RAFT end group. PMAG or p(MAG-co-FITC) (30 mg) was dissolved in 1 mL of water, followed by the addition of 100 μL of ethanolamine. Mixture was stirred at room temperature for 15 minutes. The resulting mixture was precipitated using an isopropanol/n-hexane mixed solvent (v/v = 3/2,

30 mL). The precipitate was washed with acetone (10 mL × 3) and dried under vacuum to obtain 28 mg pMAG-SH (a white solid) or 25 mg p(MAG-co-FITC)-SH (a bright yellow solid). **PMAG-A (pM-A) or p(MAG-co-FITC)-A (pMF-A)** was synthesized via a base-catalyzed Michael addition between the thiol group and the alkyne. Add 20 mg of p(MAG-co-FITC)-SH or pMAG-SH and 10 mg of AA to 100 μL of a DMSO-saturated solution of triethylamine, stir the reaction at room temperature for 12 hours, then precipitate with 30 mL of acetone. Wash the precipitate three times with acetone (10 mL × 3), and dry it under vacuum to obtain 20 mg pM-A (a white solid) or 15 mg pMF-A (a bright yellow solid).

The SEC elution curve (Figure S8) and ^{1}H NMR spectra of pMAG, pM-A, p(MAG-co-FITC), pMF-A indicate the successful synthesis of the polymers. In NMR spectra, pMAG exhibits the same peak positions as MAG, but all peaks are broad. The disappearance of the double bond peaks at 5.72, 5.66, 5.34, and 5.31 ppm indicates the successful polymerization of pMAG. Additionally, the terminal phenyl ring signals can be observed at 7.90-7.37 ppm. In contrast, pM-A shows no phenyl ring signals; instead, signals corresponding to double bonds at 8.86, 8.70, 6.90, and 6.54 ppm, as well as an alkynyl hydrogen signal at 4.14 ppm, are present. These results confirm the successful synthesis of pM-A. The spectral changes from p(MAG-co-FITC) to pMF-A are similar to those of pMAG. However, it should be noted that the phenolic hydroxyl group on the FITC residue also undergoes addition reaction with the alkyne, resulting in the appearance of four additional double bond hydrogen signals and one alkynyl hydrogen signal compared to pMAG.

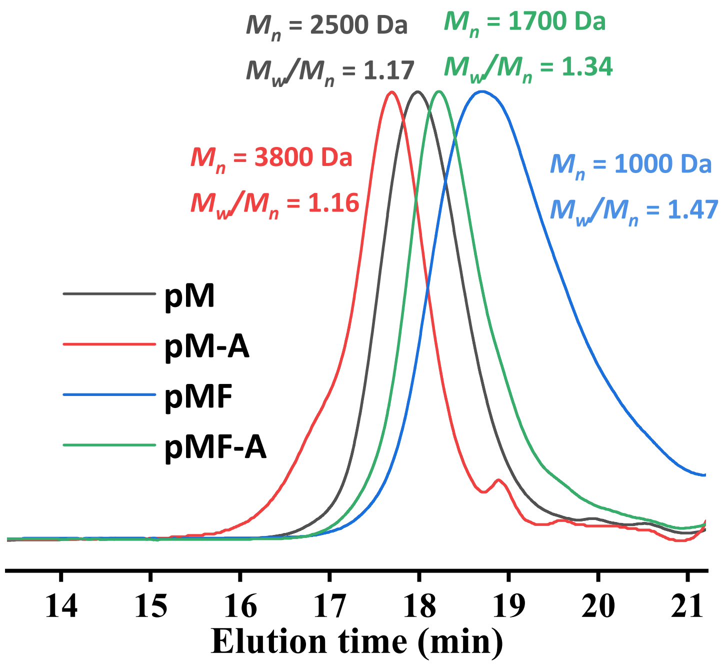

Figure S8. SEC elution curve of pM, pM-A, pMF and pMF-A

Preparation of SBA-A

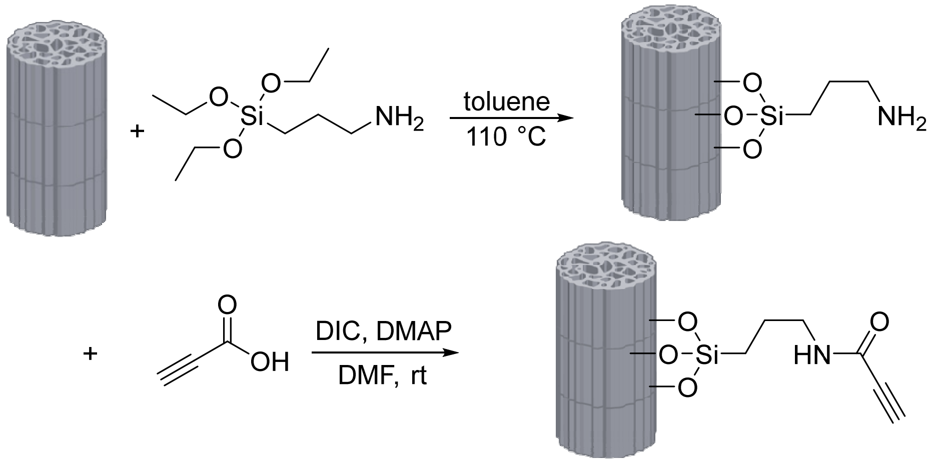

SBA-15-NH_2 was prepared by coupling amino groups onto the surface of Mesoporous silica (SBA-15) using 3-(triethoxysilyl)propan-1-amine (APTES). Mesoporous silica (SBA-15) (0.50 g), toluene (40 mL, 0.38 mol), and APTES (2.0 mL, 8.50 mmol) were added to a 50 mL round-bottom flask and stirred at room temperature for 30 minutes to ensure uniform dispersion. After refluxing at 110°C for 10 hours under condensation, the mixture was cooled to room temperature and centrifuged (10,000 rpm, 10 minutes). The obtained precipitate was washed with ethanol and dried under vacuum at 40°C. The resulting white solid (0.42 g) was named SBA-NH_2.

SBA-15-A was prepared by coupling propiolic acid (PPA) to the amino groups on the surface of SBA-NH_2 using *N, N*-diisopropylcarbodiimide (DIC) and 4-(*N, N*-dimethylamino)pyridine (DMAP). SBA-NH_2 (200 mg) was added to a mixture of PPA (64 μL, 1.03 mmol), DMAP (2.5 mg, 0.021 mmol), and DIC (130.2 mg, 1.03 mmol) in DCM (8 mL). The reaction was stirred at room temperature for 3.5 hours, then washed with DMF (10 mL × 3) and MeOH (10 mL × 3), and dried under vacuum at room temperature. The resulting pale-yellow solid (163 mg) was named SBA-A.

The FTIR characterization of SBA-15-NH_2 and SBA-15-A is shown in Section 4. Compared with the FTIR spectrum of SBA-15, SBA-15-NH_2, treated with APTES, exhibits a methylene signal at 2934 cm^{-1} and an amino signal at 1554 cm^{-1}, indicating the successful modification of amino groups on the SBA-15 surface. For SBA-15-A, the appearance of a triple bond signal at 2094 cm^{-1} and amide bond signals at 1637 cm^{-1} and 1591 cm^{-1}, in comparison to SBA-15-NH_2, confirms the successful coupling of the triple bond to SBA-15-NH_2.

Section 5. Spectra

IR Spectrum of Chol-NH$_2$

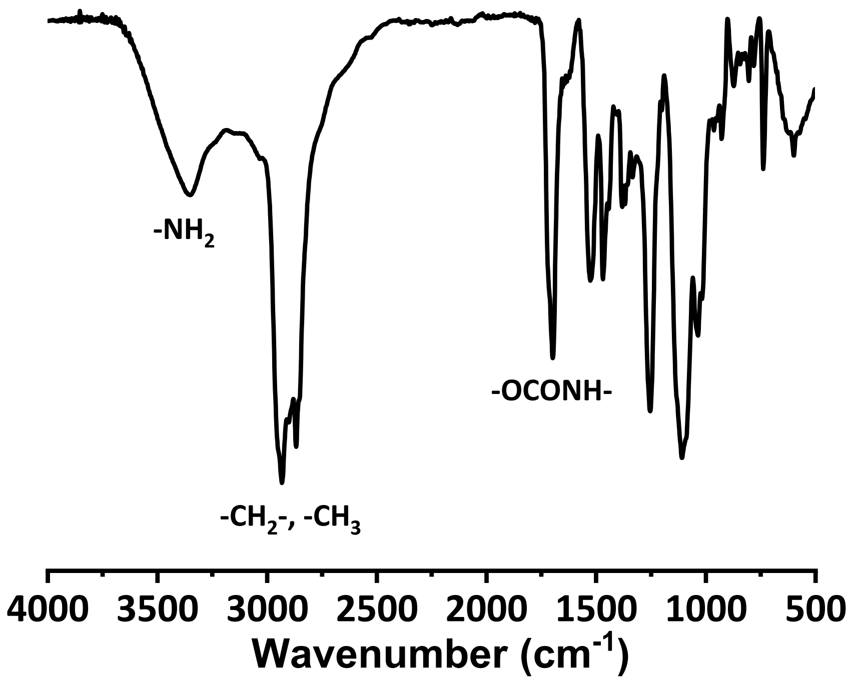

IR Spectra of SBA-15, SBA-NH$_2$ and SBA-15-A

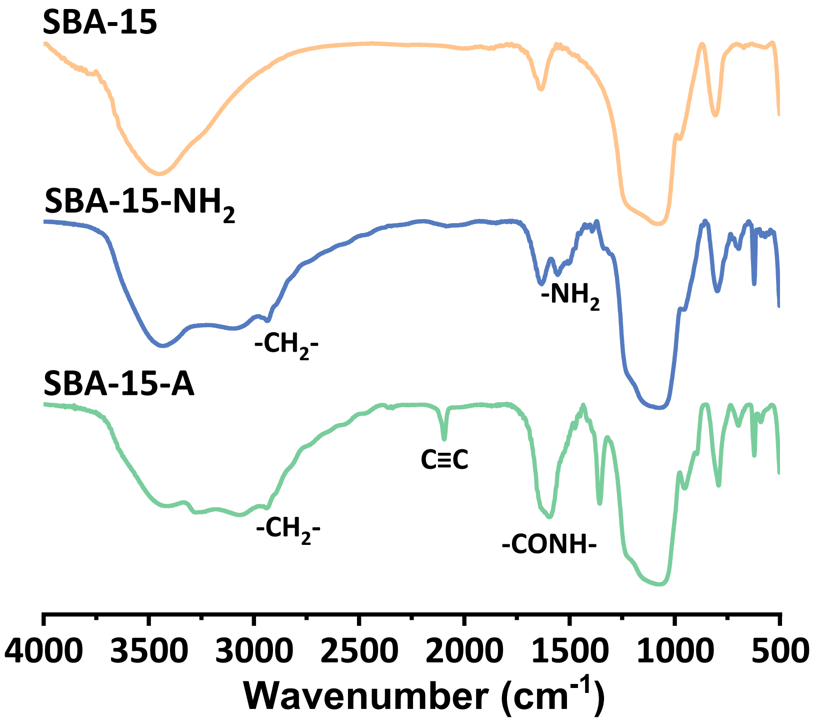

^{1}H NMR Spectrum of HSeOH (400 MHz, D_2O:DMF-d_7 = 7:1)

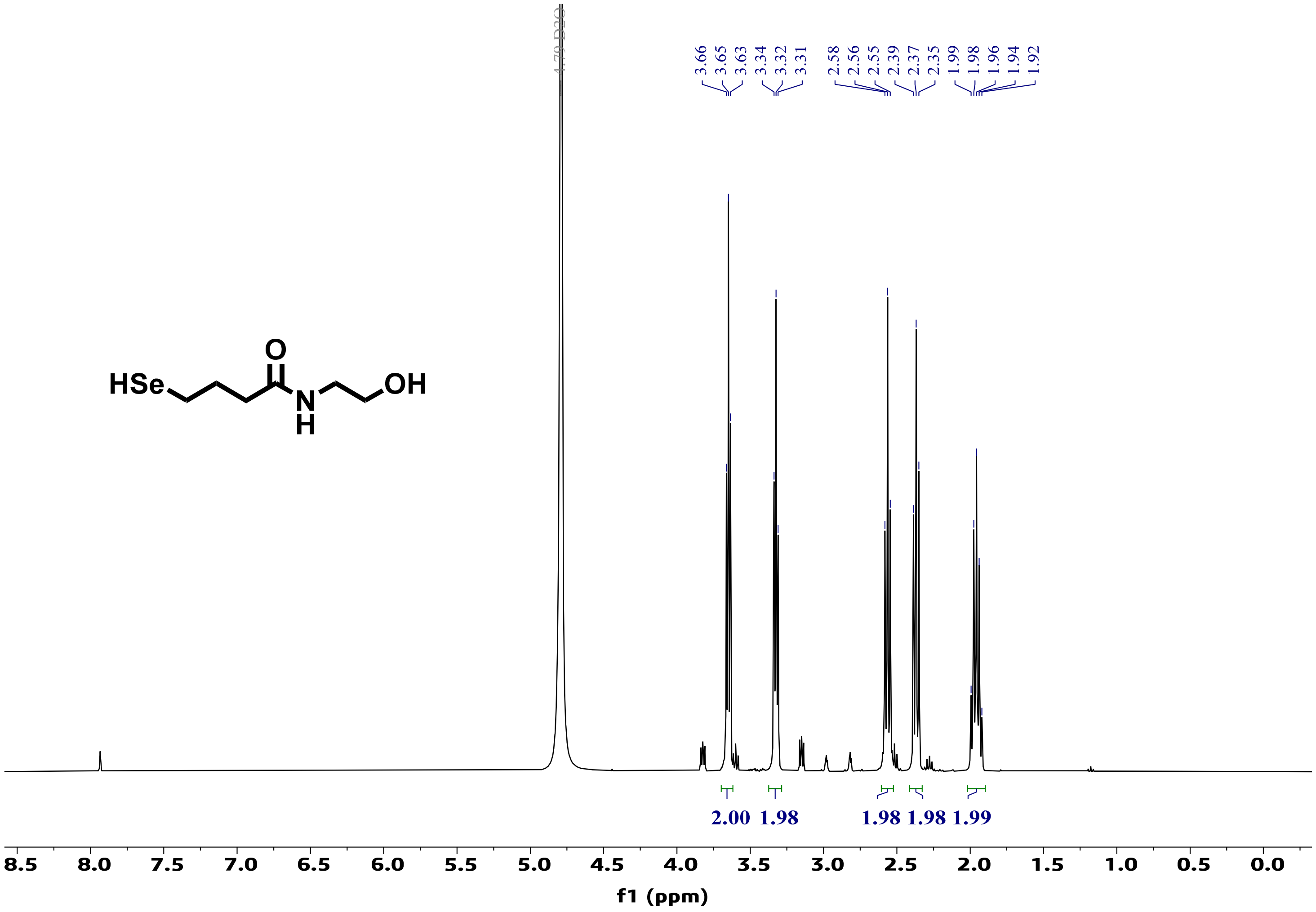

^{13}C NMR Spectrum of HSeOH (150 MHz, D_2O:DMF-d_7 = 7:1)

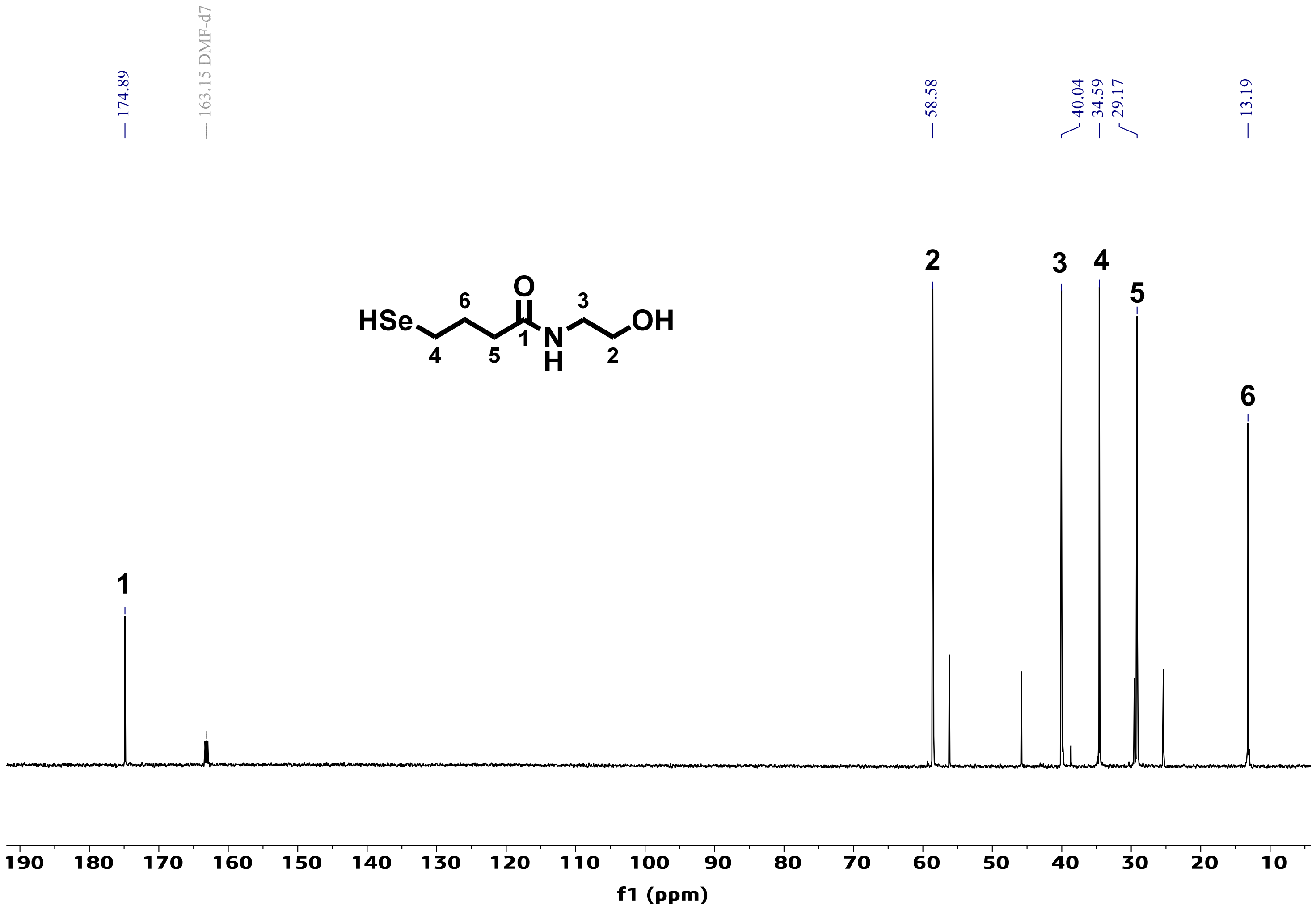

^{1}H NMR Spectrum of AA (400 MHz, DMSO-d6)

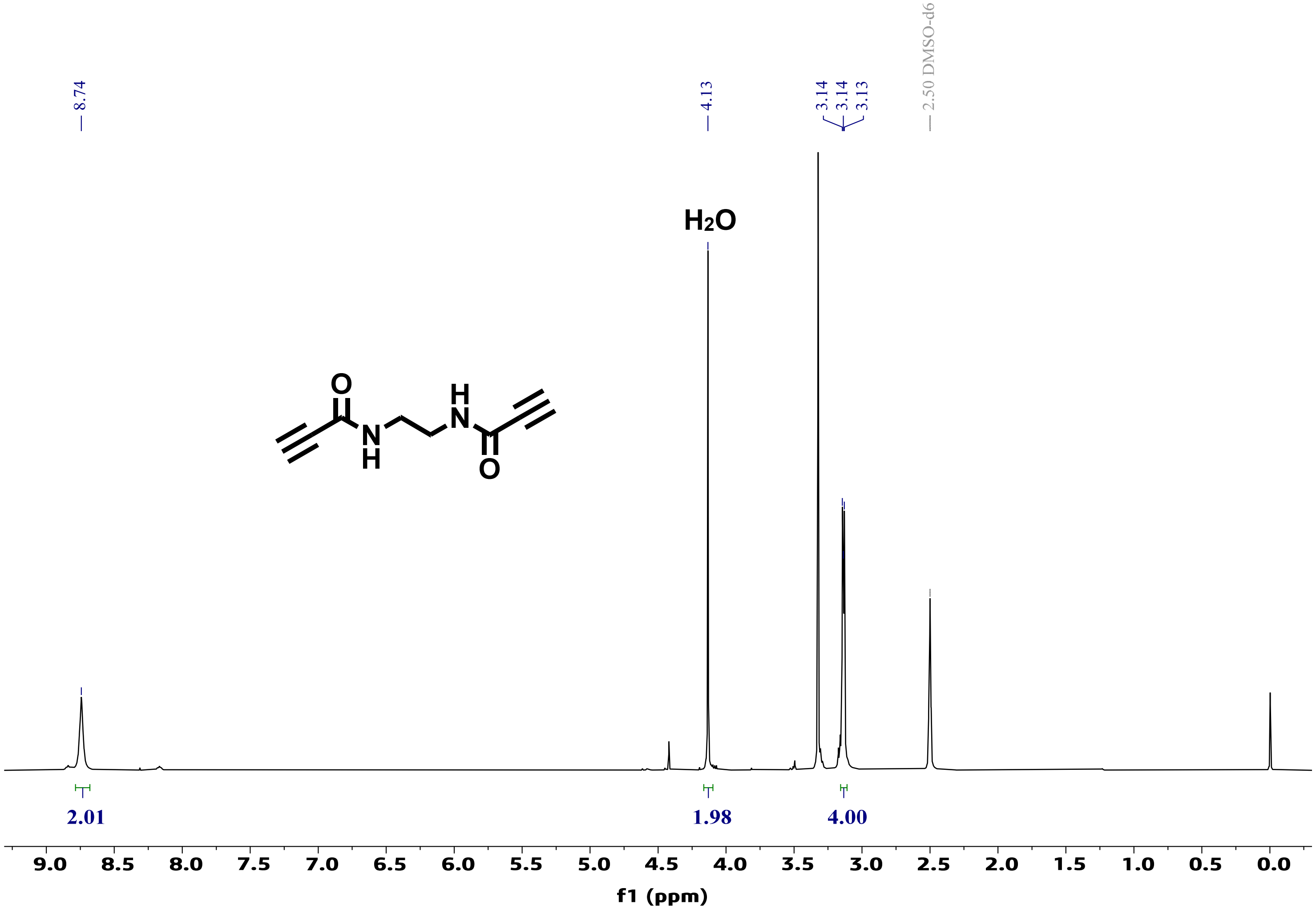

^{13}C NMR Spectrum of AA (150 MHz, DMSO-d_6)

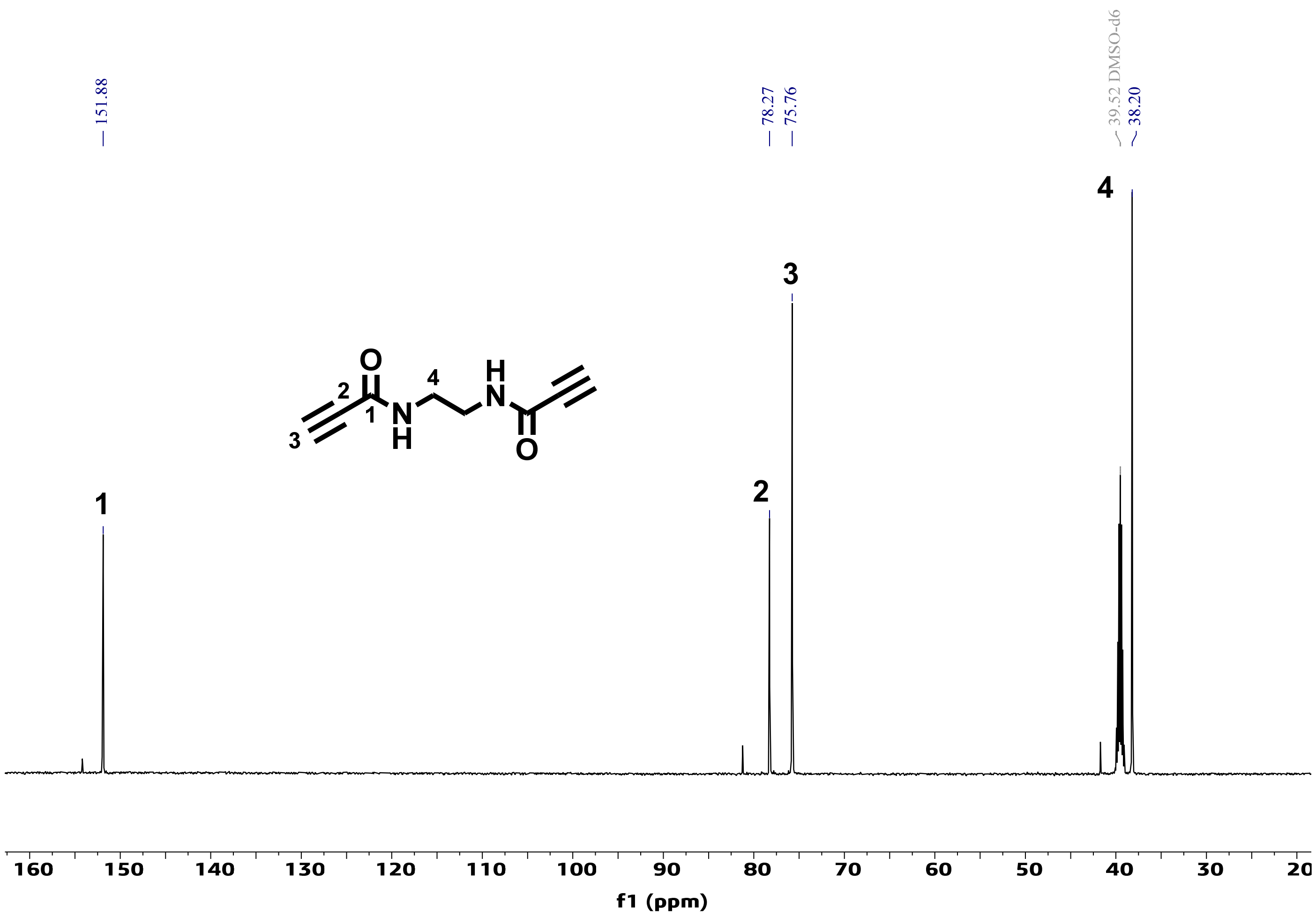

^{1}H NMR Spectrum of DSeOH (400 MHz, D_2O:DMF-d_7 = 7:1)

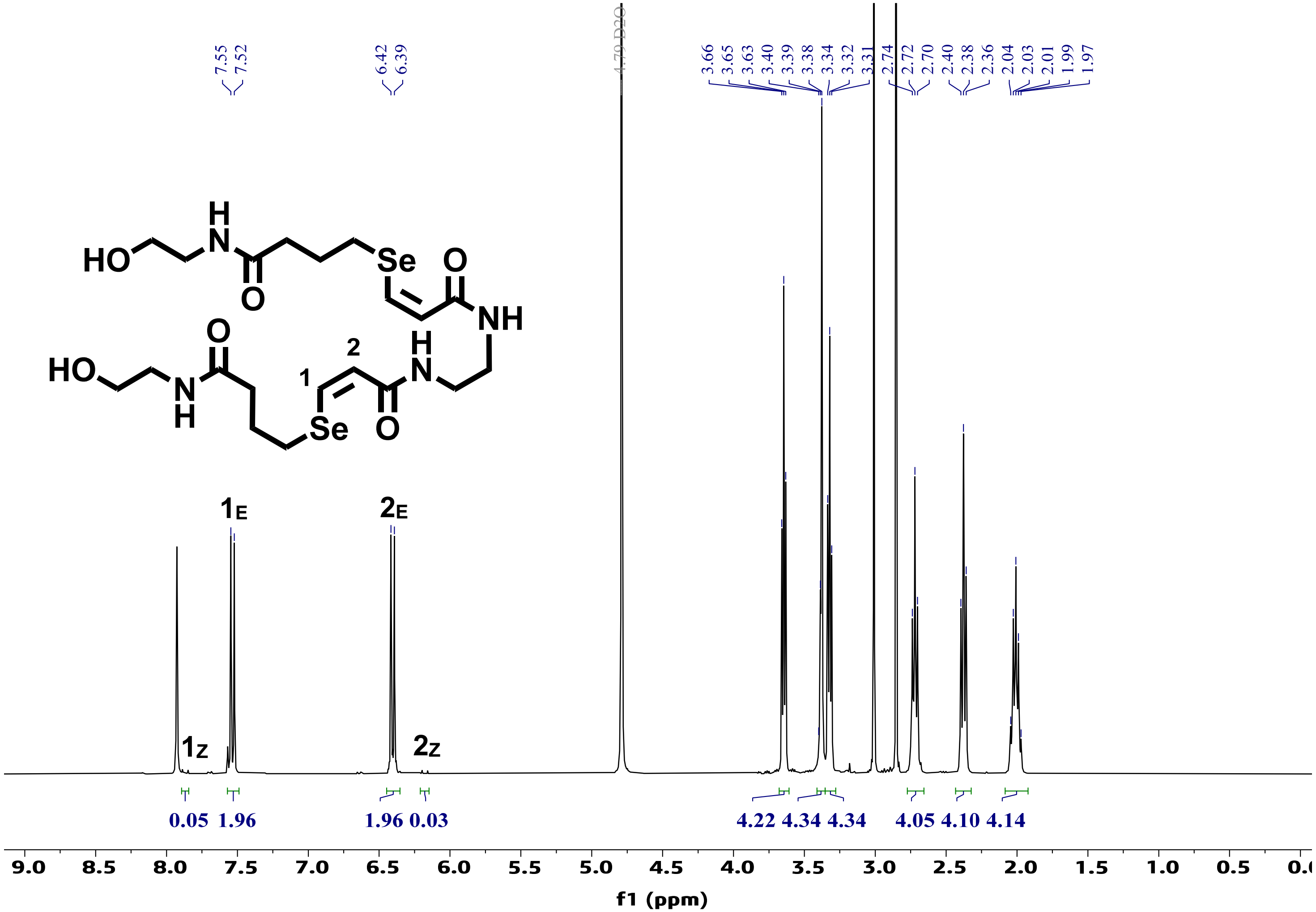

^{13}C NMR Spectrum of DSeOH (150 MHz, D_2O:DMF-d_7 = 7:1)

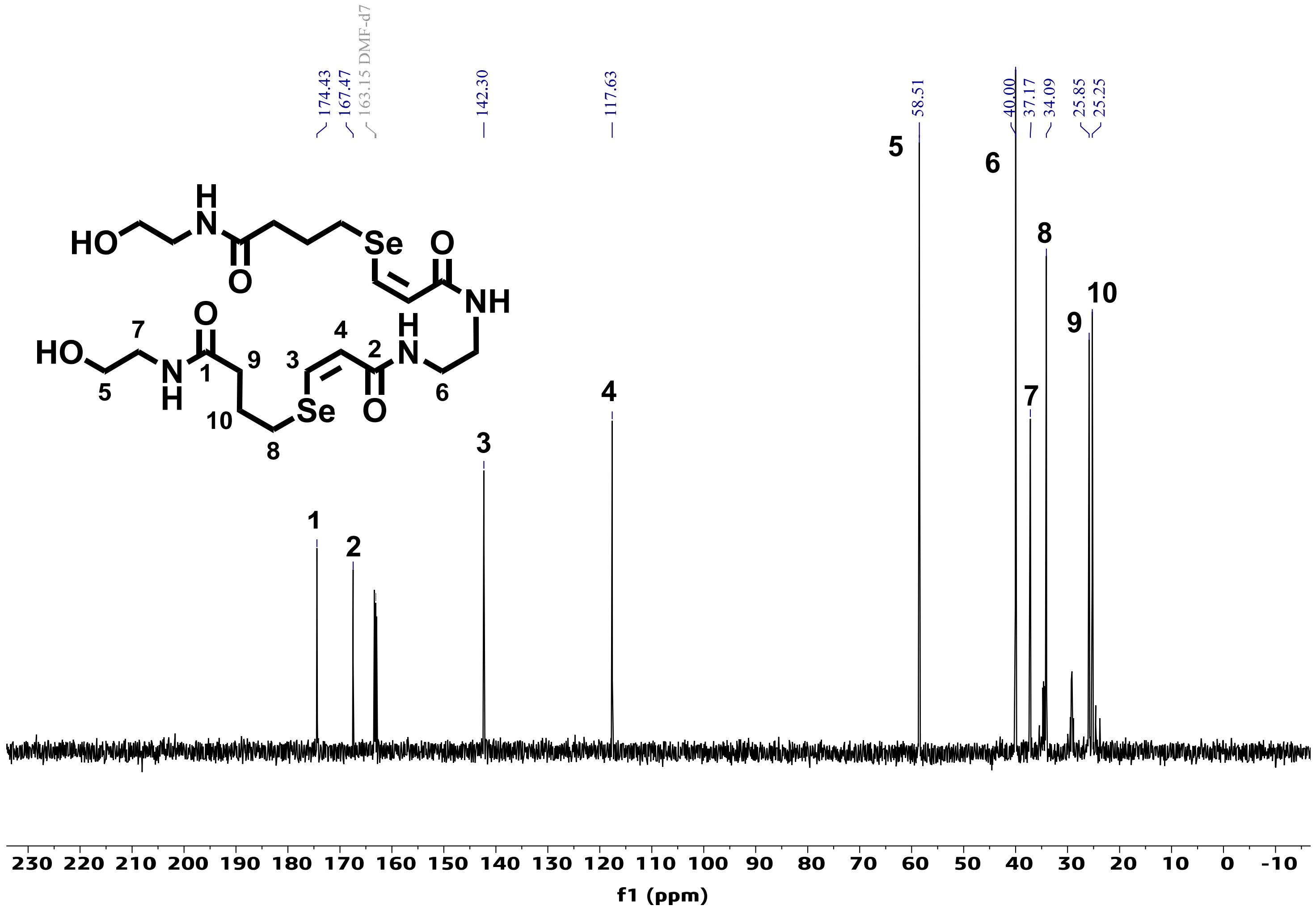

^{1}H NMR Spectrum of Chol-NH$_2$ (400 MHz, CDCl$_3$)

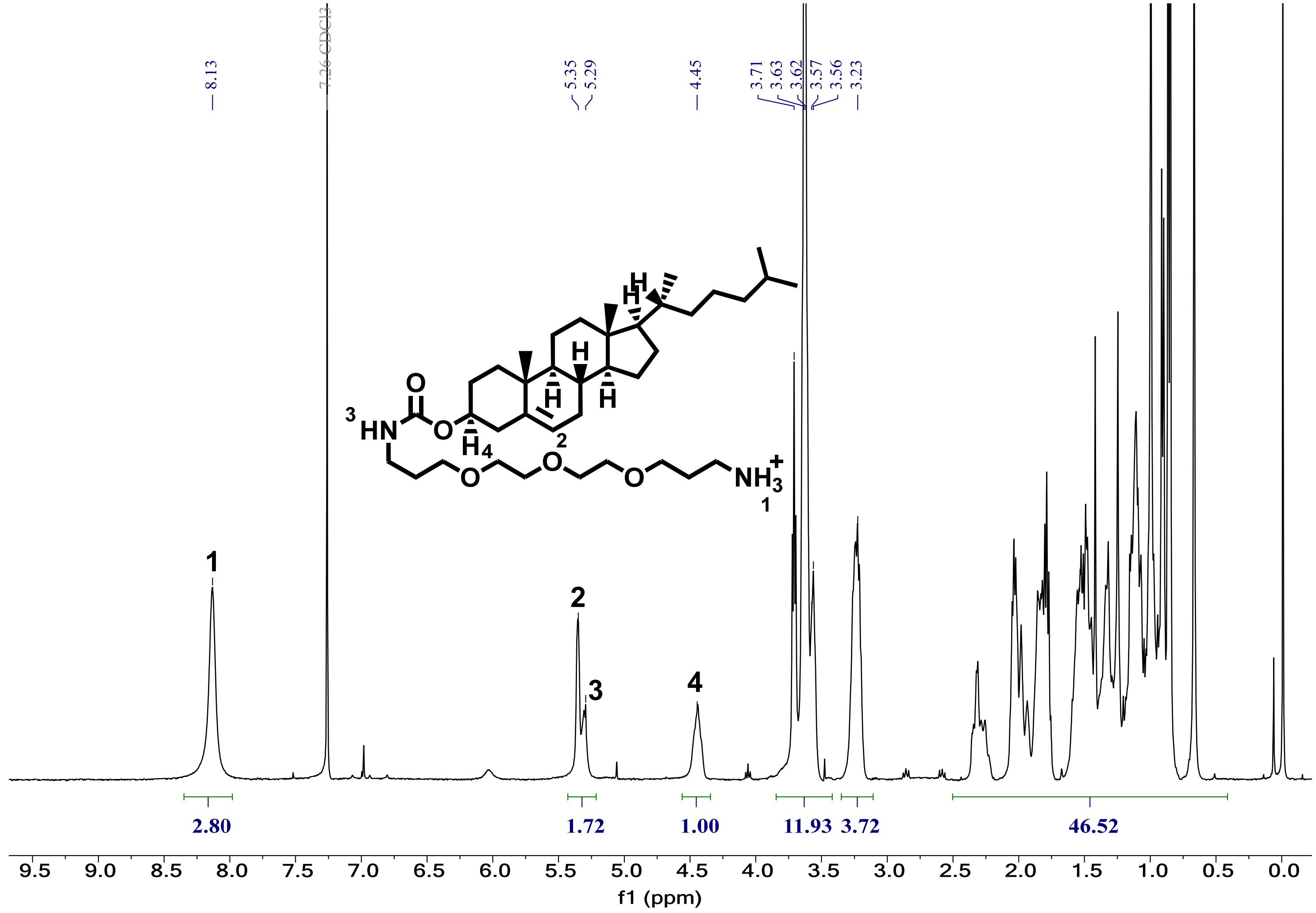

^{13}C NMR Spectrum of Chol-NH$_2$ (150 MHz, $CDCl_3$)

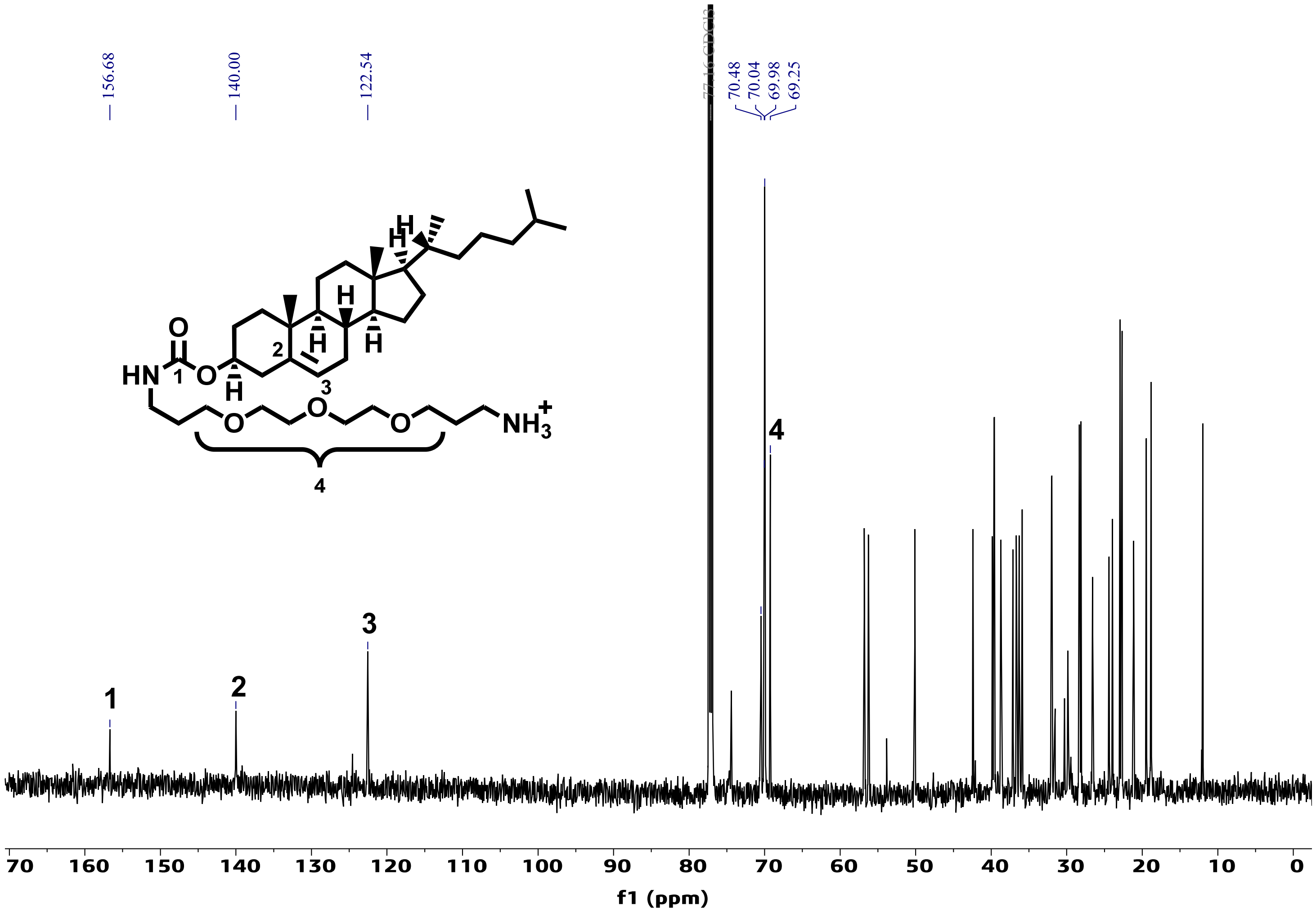

^{1}H NMR Spectrum of Chol-SeH (400 MHz, $CDCl_3$)

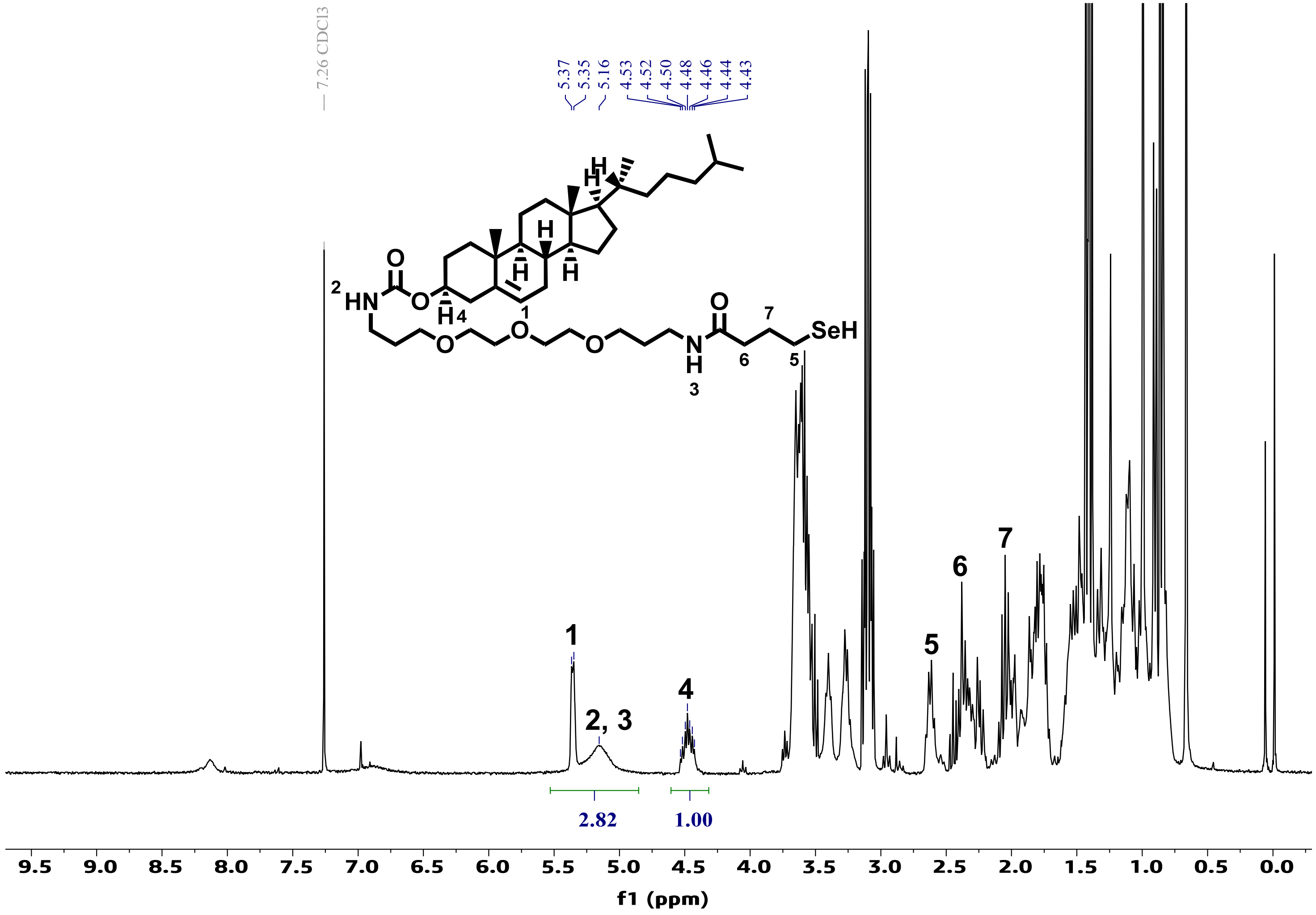

^{13}C NMR Spectrum of Chol-SeH (150 MHz, $CDCl_3$)

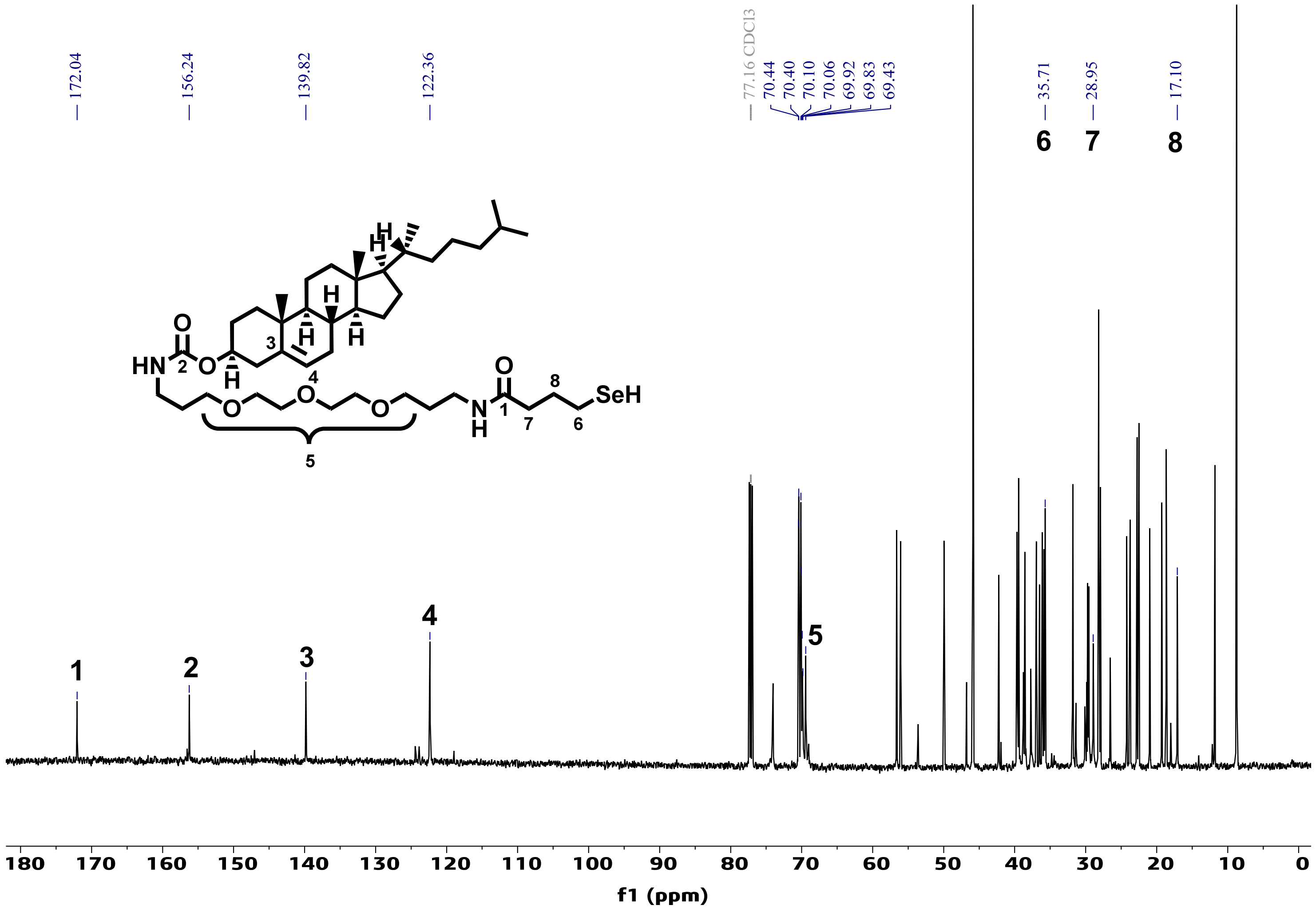

^{1}H NMR Spectrum of FITC-A (400 MHz, $CDCl_3$)

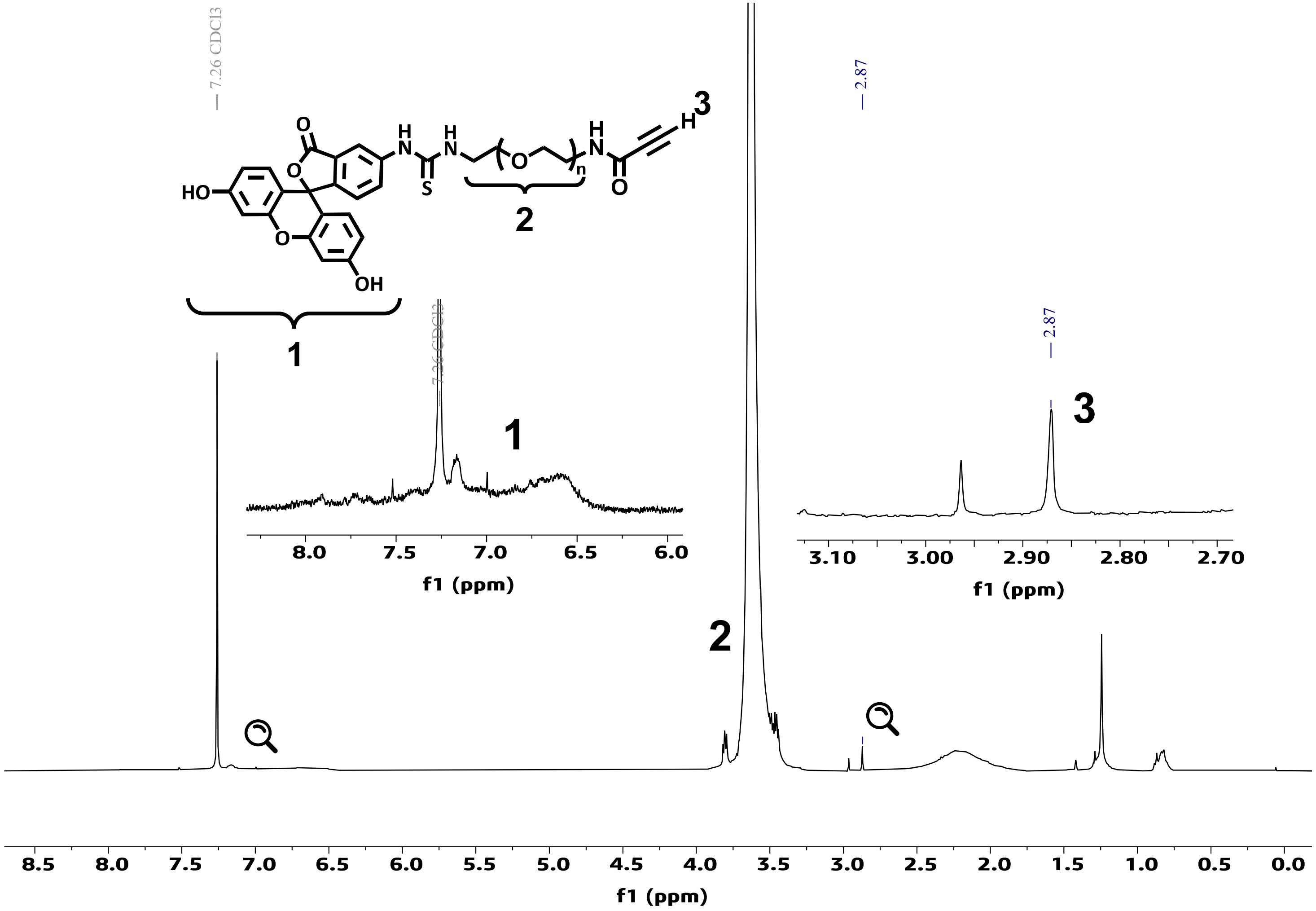

^{1}H NMR Spectrum of mPEG-A (400 MHz, $CDCl_3$)

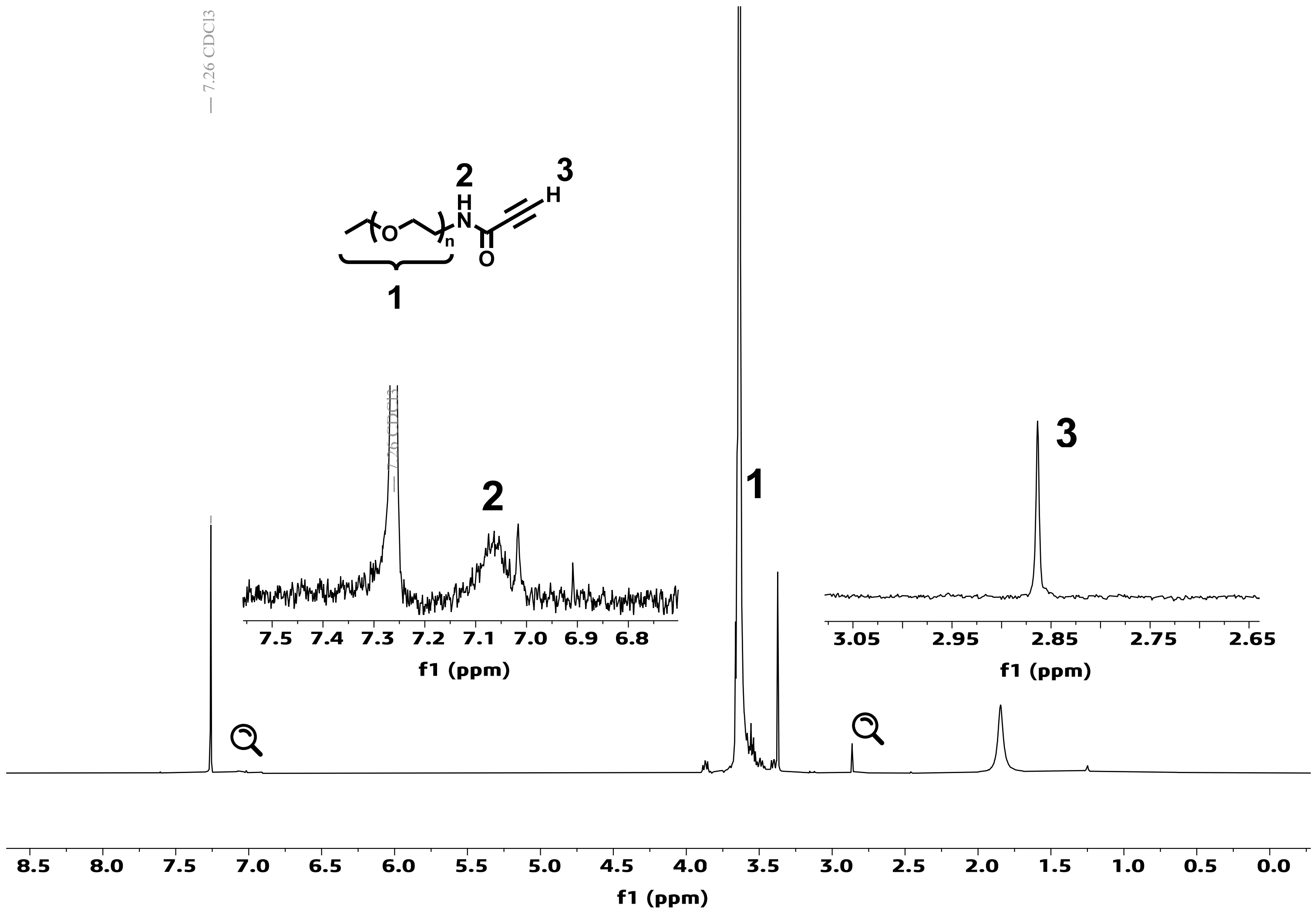

^{1}H NMR Spectrum of MAG (400 MHz, DMSO-d6)

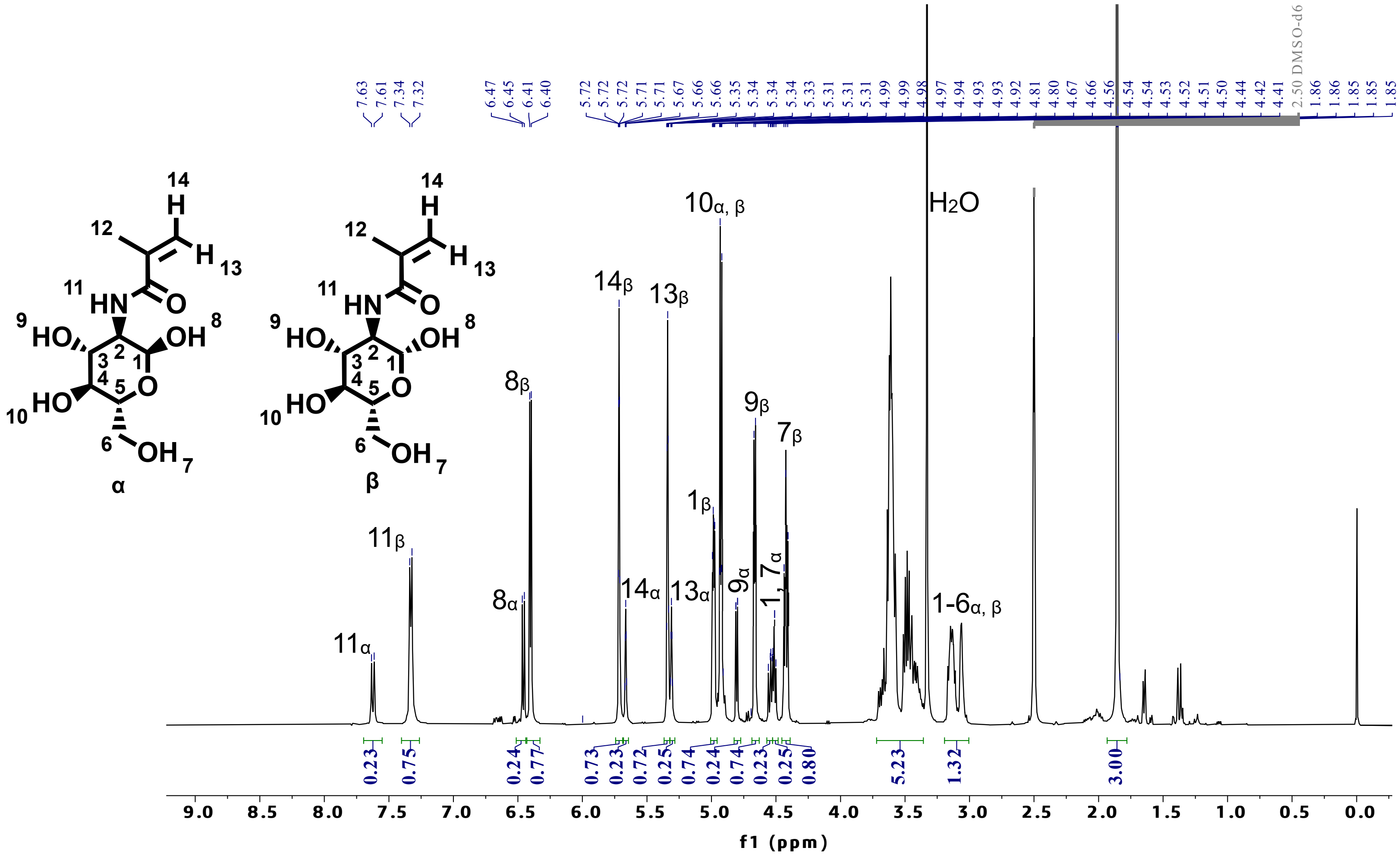

^{1}H NMR Spectrum of MAG (300 MHz, D_2O)

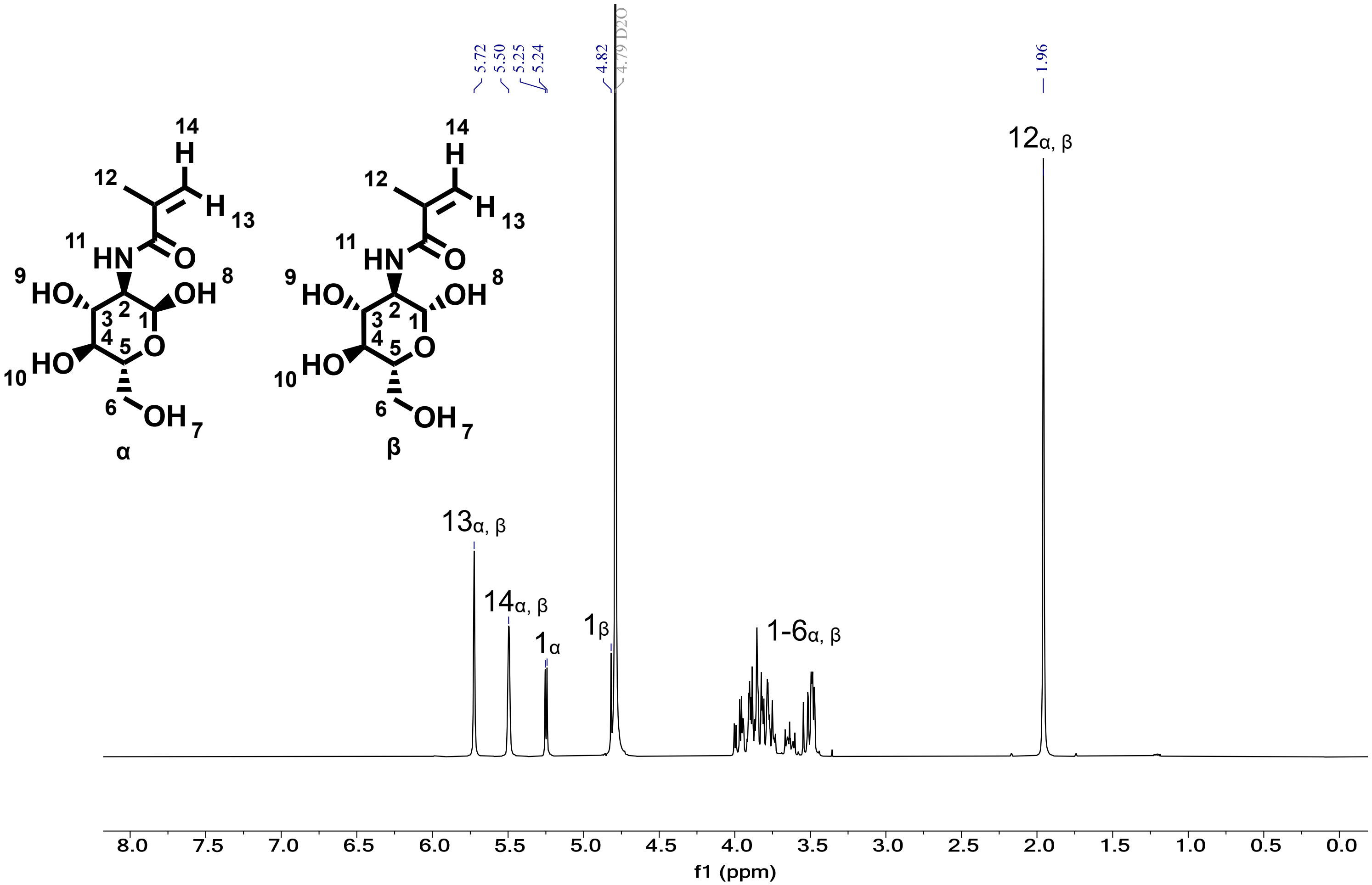

^{1}H NMR Spectrum of pM, pM-A, pMF and pMF-A (400 MHz, DMSO-d_6)

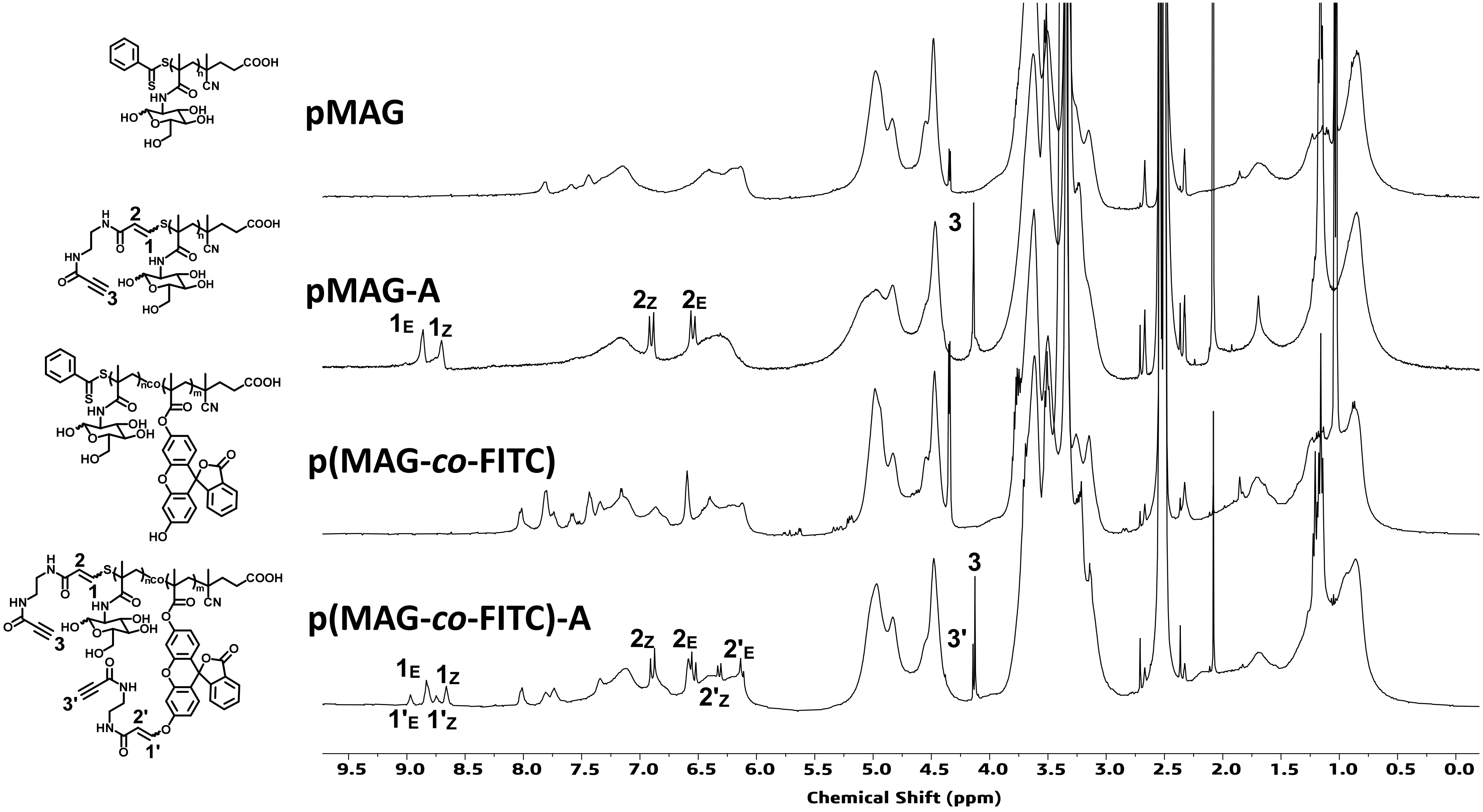

HRMS Spectrum of AA

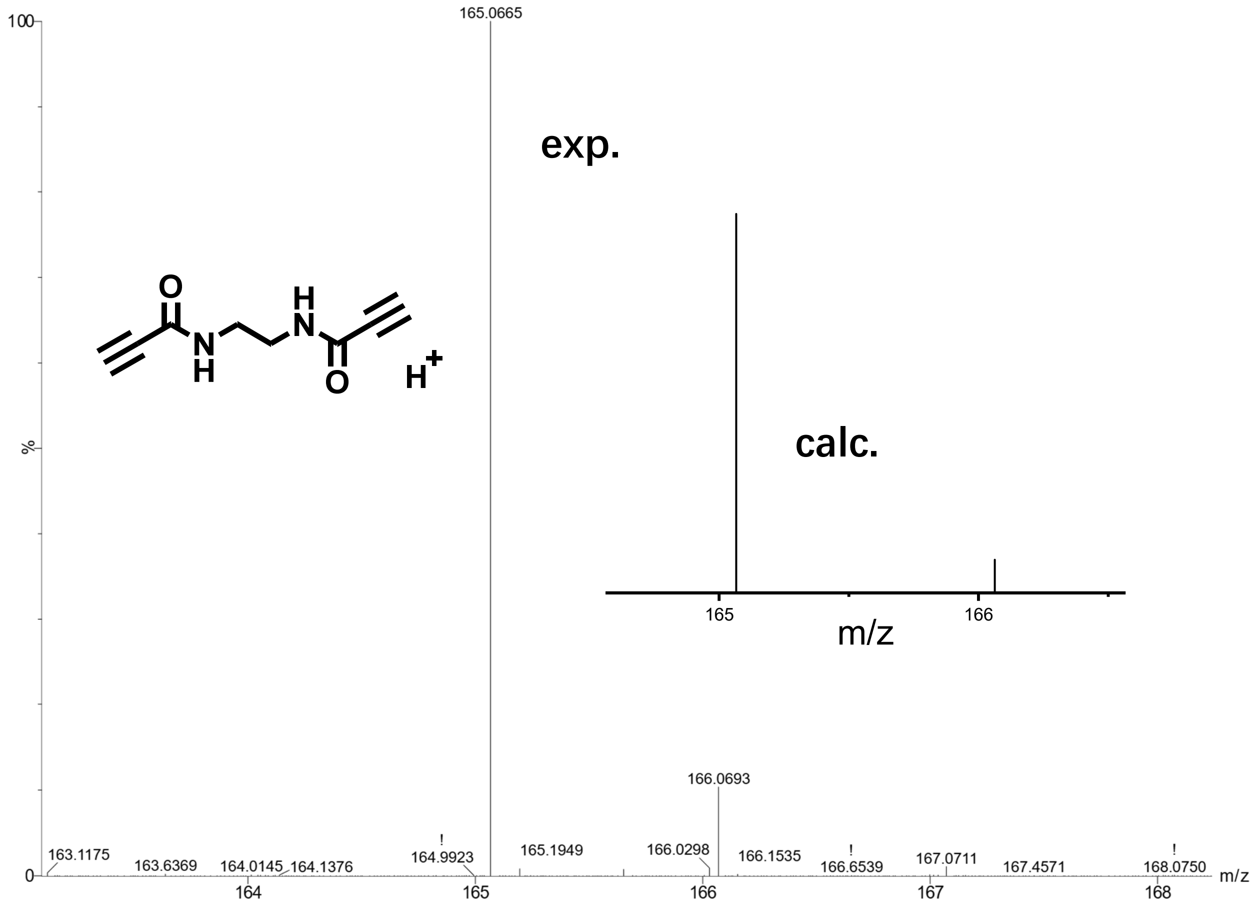

HRMS Spectrum of HSeOH

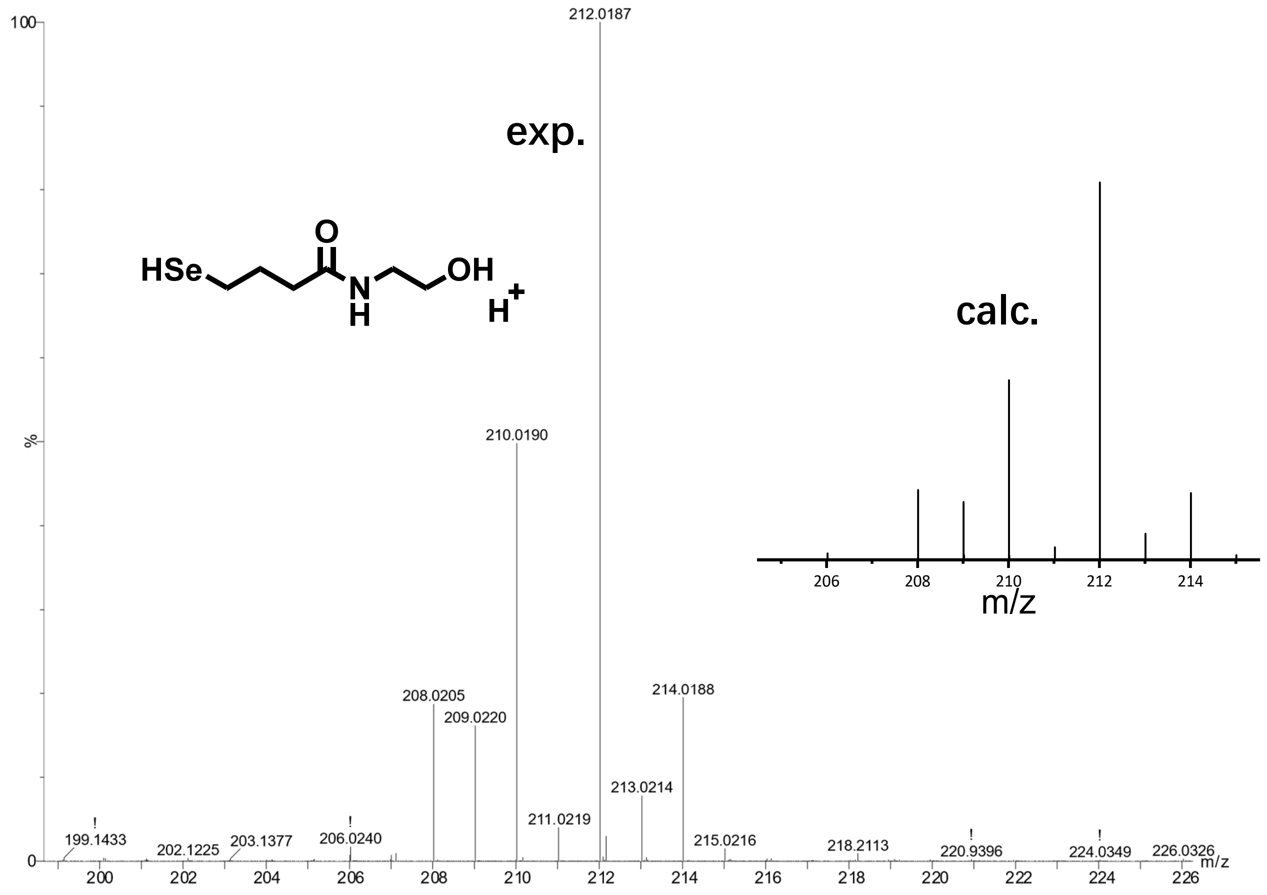

HRMS Spectrum of DSeOH

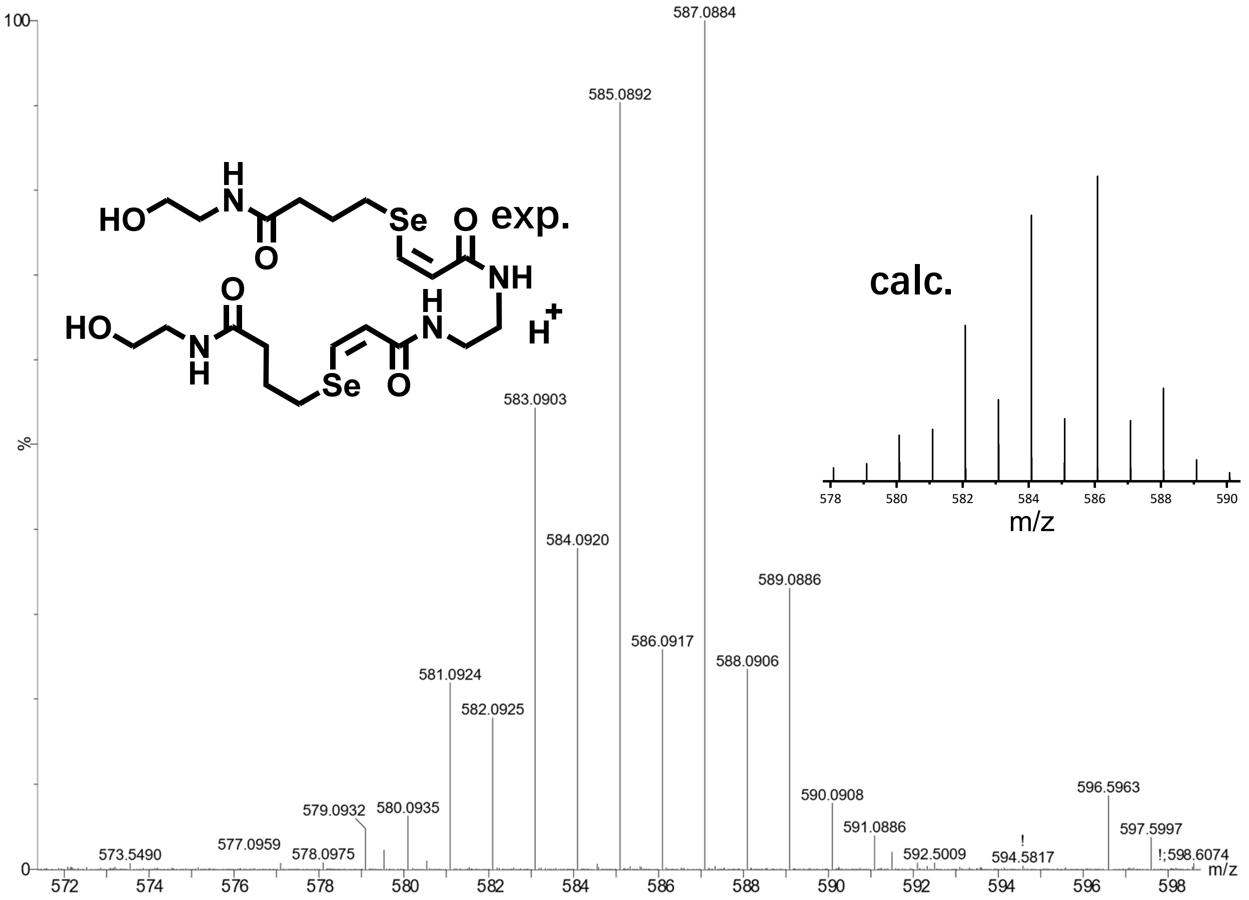

HRMS Spectrum of Chol-NH_2

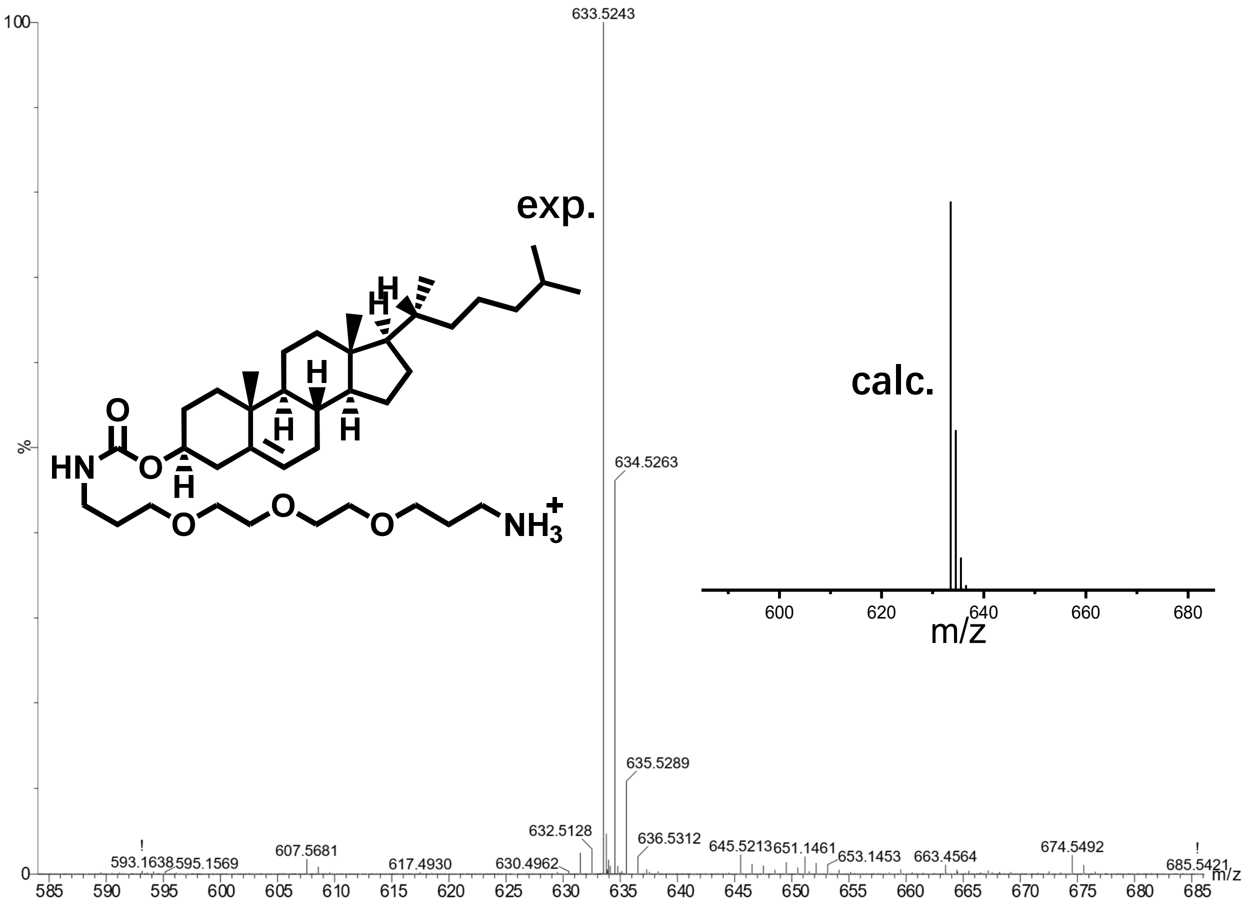

HRMS Spectrum of Chol-SeH

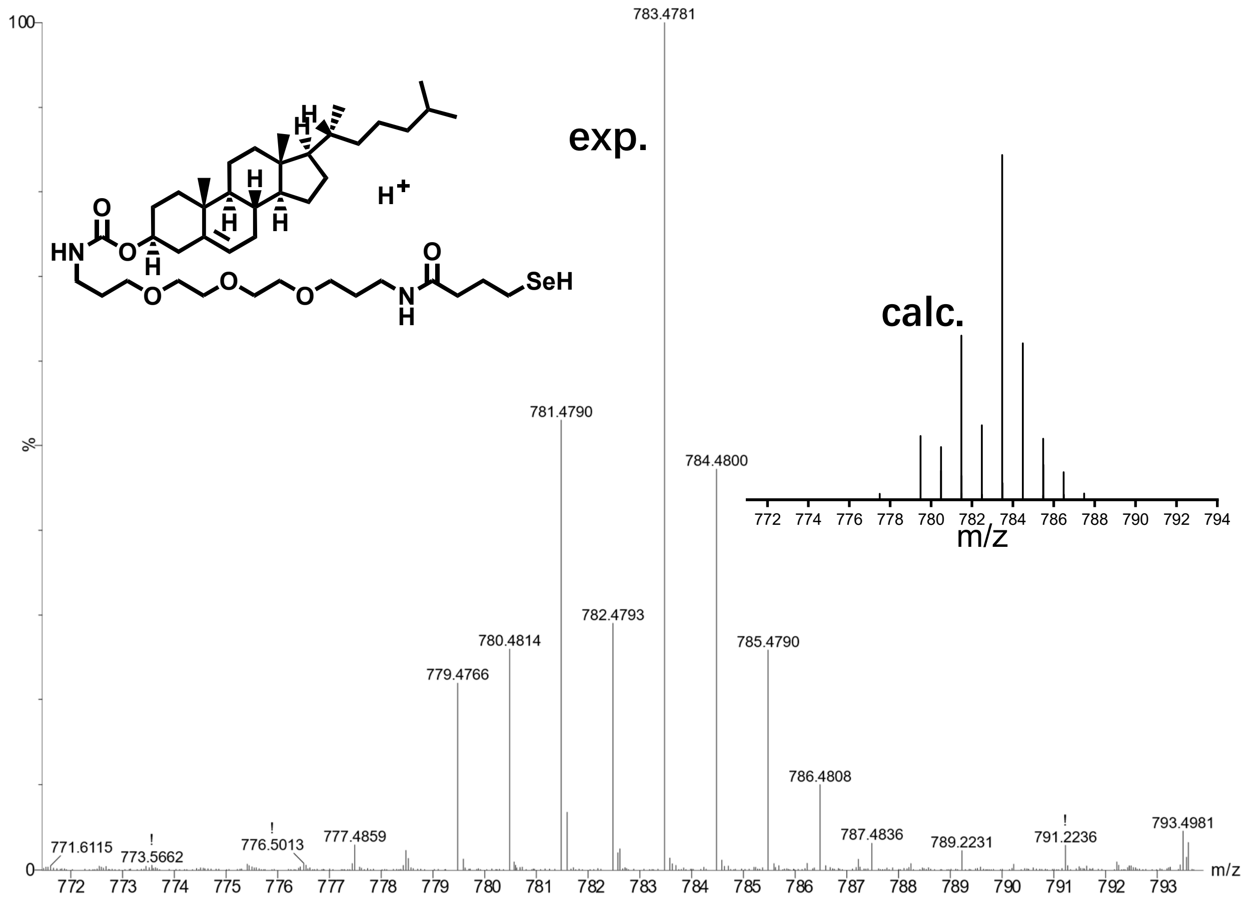

References

1 X. Heng, F. Shan, H. Yang, J. Hu, R. Feng, W. Tian, G. Chen and H. Chen, *Adv. Healthcare Mater.*, 2023, **12**, 2301536.

2 S. Pearson, N. Allen and M. H. Stenzel, *Journal of Polymer Science Part A: Polymer Chemistry*, 2009, **47**, 1706-1723.

3 Y. Hong, W. Zhong, M. Zhang and H. Zhao, *Macromolecules*, 2021, **54**, 11238-11247.
